# Study of a Local Structure at the Interface between Corrosion Films and Carbon Steel Surface in Undersaturated CO_2_ Environments

**DOI:** 10.1021/acsomega.2c07631

**Published:** 2023-02-23

**Authors:** Adriana Matamoros-Veloza, Tomasz M. Stawski, Silvia Vargas, Anne Neville

**Affiliations:** † Faculty of Engineering and Physical Sciences, University of Leeds, Leeds LS2 9JT, United Kingdom; ‡ Institute of Functional Surfaces, School of Mechanical Engineering, University of Leeds, Leeds LS2 9JT, United Kingdom; § Federal Institute for Materials Research and Testing (BAM), Richard-Willstatter-Straße 11, 12489 Berlin, Germany; ∥ BP America, Inc., Houston, Texas 77079, United States

## Abstract

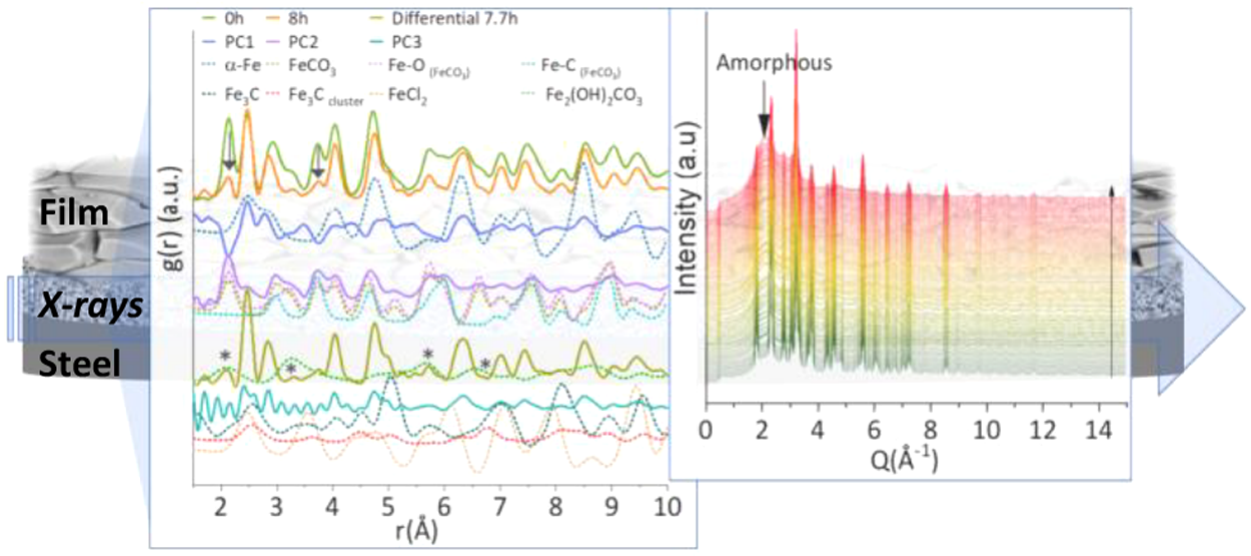

Industries transporting CO_2_ gas-saturated fluids have infrastructures made of carbon steel. This is a good material with great mechanical properties but prone to corrosion and potential failure. Corrosion in sweet environments involves the formation of FeCO_3_ as a corrosion film, which is recognized to play a protective role under certain conditions. This work on the dissolution of corrosion films in sweet environments, under acidic and undersaturated conditions, demonstrates that the effects on the integrity of steel are far more significant than the damage observed on the surface of the corrosion film. Our results prove that dissolution of FeCO_3_ involved the presence of an amorphous phase, the intermediate formation of FeCl_2_ or FeCl^+^, and the presence of a phase with short distance atom–atom correlations. The amorphous phase was identified as a mixture of retained γ-Fe and Fe_3_C. Partially broken α-Fe and Fe_3_C structures were identified to prove the damage on the material, confirming the interface zone without evident damage on the corrosion film. Dissolution affected both the α-Fe and FeCO_3_, with the lattice [102̅] from the FeCO_3_ crystalline structure being the fastest to dissolve. The damage of steel at the molecular scale was evident at the macroscale with pit depths of up to 250 μm. The impact on the integrity of steel can be, therefore, more drastic than frequently reported in industrial operations of CO_2_ transport industries that use cleaning procedures (e.g., acid treatment, pigging) as part of their operational activities.

## Introduction

Moving fast toward net-zero carbon emissions, innovative approaches for carbon dioxide capture need to be adopted at the industrial scale to meet the targets set at the Paris Agreement.^1^ Industries which transport fluids saturated with CO_2_ gas have their infrastructures made of carbon steel (e.g., pipes, tanks, pumps, etc.). The convenience of carbon steel, apart of its low cost, lies in its favorable mechanical properties, such as strength, toughness, bearing stress, impact resistance, ease of welding, and thermal processing.^2^ Despite the benefits of using carbon steel for the transportation of CO_2_, this material is prone to corrosion, leading to multiple adverse and serious consequences such as downtime in plant operations and risks at various levels: operators, community, and environment.^3−6^ Beyond these, the failure of carbon steel infrastructure involves excess costs to ameliorate damages. For example, in the U.S. only, major losses related to pipeline failure incidents’ damage costs are reported to amount to U.S. $6.9 billion between 2010 and 2020. The 6950 incidents totaled 688 injuries, 156 fatalities, 43,803 evacuees, 1005 fires, and 355 explosions which rationalizes the need for a deeper understanding of the material for CO_2_ transport, among other applications.^7^

Corrosion of carbon steel has been reported as either or both general and localized processes. General corrosion involves the precipitation of iron minerals on the steel surface consuming iron from ferrite (α-Fe) and pearlite (ferrite,α-Fe; and cementite, Fe_3_C), which are the main components of the steel microstructure. The most common corrosion product forming under CO_2_ environments (so-called “sweet environments”) is siderite (FeCO_3_), although oxides and oxyhydroxides may also form depending on conditions of pH, temperature, pressure, and air presence.^8−10^ At the initial stages of corrosion formation, elements are consumed away from the steel surface (i.e., initial steel degradation). But it is overall believed that the formed corrosion layer impedes the entrance of further corrosive species, hence the recurrent positive designation of siderite (FeCO_3_) as a protective corrosion layer. On the other hand, the stability of FeCO_3_ depends on various environmental and physicochemical conditions, such as temperature, pH, partial pressure of CO_2_, flow velocity, and steel microstructure.^3,11,12^ In addition, underneath the thick siderite corrosion layer (typically ∼50 μm from laboratory studies), a thin layer composed of fine material is commonly reported, but the nature and composition of this material are yet to be characterized.^8−10^

Cementite (Fe_3_C) is also frequently mentioned to be present as an undissolved phase after the dissolution of α-Fe from steel to form the FeCO_3_ corrosion film.^8,13−19^ The resulting Fe_3_C phase consists of a porous network that either is in close contact or is intermingled with the FeCO_3_ corrosion film. Various authors suggest that Fe_3_C increases corrosion rates because of its ability to form a galvanic connection with α-Fe, promoting local acidification.^14,20−22^ Despite all these observations, other works have suggested a beneficial role of Fe_3_C as inductors of the “protective” FeCO_3_ formation through the solubilization of α-Fe (Fe^2+^ release) to form FeCO_3_.^22^ However, the porous nature of Fe_3_C and the suggested local acidification effect of this process can counteract any potential protective benefits of FeCO_3_ corrosion films.^20^ Localized corrosion (pitting) is a more aggressive and stochastic type of corrosion causing the formation of voids up to 150 μm in depth on the steel surfaces. However, this type of corrosion is poorly understood, and its origins are still a matter of intensive research.^23,24^

The above presented literature review highlights the need of further research to understand the corrosion processes at the interface, i.e., a zone where steel and corrosion products interact. Needless to say, this is a challenging task, and few works have made attempts to study this zone using only ex situ electrochemical techniques. Farelas et al.^14^ studied the dissolution of corrosion products at the interface using impedance spectroscopy and linear polarization resistance to quantify electrochemical information, which then was used to infer the nature of corrosion processes. But this work did not include structural characterization methods, such as X-ray diffraction, Raman spectroscopy, or electron microscopy techniques, to support the interpretation. To fill this knowledge gap, in this work, we identify the corrosion products and their transformations at the interface during the dissolution of a corrosion FeCO_3_ film (under saturated conditions) using in situ high-energy X-ray diffraction and pair distribution function analysis (HEXD/PDF). Innovatively, we applied this method for the first time in this field, which allowed the direct quantification of the chemical changes at an atomistic level that reliably led us to a better understanding of the corrosion processes. These techniques provided us with the information on the local structure of the crystalline and/or amorphous materials, as the dissolution process progressed. The results of this work offer further understanding of the behavior of steel material under conditions and operations encountered in CO_2_ transport industries (e.g., acid treatment, pigging) contributing to the evaluation of the integrity and potential risks of failure of this material.

## Experimental Section

### Synthesis Procedure

Manufactured carbon steel (X65) cylindrical specimens with a diameter of 10 mm and a height of 6.25 mm were designed with a landing step of 3 mm wide and a step height of 5 mm in top hat geometry (Figure S1). Each specimen had a total surface area of ∼5.49 cm^2^. On the base of the specimen, a centered hole was tapped for holder mounting. C-steel specimens were polished using progressively 120, 320, and 600 silicon carbide (SiC) grit papers, then rinsed with DI water and acetone, and dried with air.

For corrosion film growth, the specimens were attached to a custom-made holder placed inside of an autoclave reactor and fixed to the shaft of the autoclave lid. An unstirred 1% NaCl solution, saturated with CO_2_ for 24 h, was placed in the autoclave, and the pH was adjusted to 7.0 at 80 °C using NaHCO_3_. Thus, corrosion films formed in a static mode at 80 °C under a CO_2_ total pressure of 30 bar for 72 h.^13^ Corroded specimens were rapidly removed from the autoclave, rinsed with DI water, dried with air, and transferred immediately to a vacuum desiccator. The specimens were kept in a vacuum inside sealed bags until they were used in flow experiments or used for characterization. Chemicals used in the formation of corrosion films were NaCl (Fluka/Honeywell CAS 7647-14-5 99%) and NaHCO_3_ (Alfa Aesar 99% CAS 144-55-8).

### Dissolution of Corrosion Films

Dissolution occurring at the interface between corrosion products and steel was evaluated under a turbulent flow velocity of 1 m/s, 1% NaCl at room temperature, and 80 °C. The pH of the CO_2_-saturated brine solution was ∼3.6 or 3.3 (HCl addition). The pH was constantly monitored throughout the reaction. We used a recirculated custom-built flow cell system controlled with a high precision magnetic drive gear micropump (micropump series GJ-N25) consisting of a reactor vessel assembled to maintain strict CO_2_-saturated atmosphere fitted with temperature and pH probes and with inlet and outlet ports, as shown in Figure S1 and described in detail in elsewhere.^13^

### Characterization of Corrosion Films and Steel Surface

Immediately after removing the as-formed specimens, they were characterized using X-ray diffraction (XRD) and scanning electron microscopy (SEM). For the XRD analysis, the specimens were mounted onto a holder and scanned from 15 to 80° 2θ at 1.55°/min using a Bruker D8 X-ray diffractometer. The diffraction patterns were compared against the diffraction data of the structure of FeCO_3_ to confirm its formation as a main corrosion product.^25^

For SEM imaging acquisition, a TM3030Plus microscope was used. The specimens were fixed onto stubs using high-purity double-sided conductive adhesive carbon tabs and mounted on the sample stage of the instrument which was operated at 15 kV. After dissolution experiments at the beamline, the specimens were rapidly removed from the flow cell, rinsed with DI water, dried immediately with air, and then stored in a vacuum desiccator for further SEM imaging. We used interferometry to map pits over an area of 5.0 mm × 4.0 mm of the steel surface after the dissolution reaction at 80 °C using a NPFLEX 3D Bruker interferometer; profiles of the diameter and depth of the pits were recorded during the analysis.

### Total Scattering

X-ray total scattering data were collected at the I15-1 beamline at the Diamond Light Source, U.K., using λ = 0.161669 Å (76.7 keV). Measurements were taken for a 2θ angular range between 0.02 and 60° corresponding to a *Q* range of 0.01–40 Å^–1^ (*Q*_max_ = 4π sin θ/λ). A flow cell setup containing a previously corroded specimen (top hat geometry) was exposed to the beam (Figure S1). Experimental conditions were pH 3.6, 80 °C, 1 m/s, 1% NaCl. The position of the sample was calibrated with a CeO_2_ standard, and the collected 2D X-ray scattering data were processed into 1D patterns using DAWN software.^26^

To calculate the PDFs from the diffraction data, the patterns were corrected for background (using equivalent measurements taken from a flow cell with brine solution with no steel sample), multiple scattering, container scattering, Compton scattering, and absorption, which were all performed using the GudrunX program.^27,28^ The extracted PDFs represent contributions from all atom–atom correlations in the system. To distinguish the more common distinct structural elements in the series of data, principal component analysis (PCA) was performed in Origin Pro software^29^ with normalized PDFs. Significant components of the PCA can be related to the distinct phases, species, and structures in the system,^30^ and the relative contribution of the components to each processed PDF provides an indication of how these vary within a series related to the change in relative concentration of the different constituents. PDF simulation was performed in PDFGui.^31^

Rietveld refinement using a model to include FeCO_3_, α-Fe, and FeCl_2_ was performed in TOPAS v.6.29^32^ to fit the data within the range of 1.5–40 Å. Bragg data were imported and normalized for the calculation of the integral breadth (area/maxima intensity) to evaluate the change of breadth of the [102̅] reflection over time. The position of this peak was evaluated for the duration of the experiment.

## Results and Discussion

### Synthesis of Corrosion Sales on Carbon Steel

Corrosion film grown on a carbon steel surface was confirmed to be FeCO_3_ by comparing the diffraction data collected from the corrosion film with siderite from the reference database (Figure S2a, ICCD 8-0133).^25^ In agreement with our previous work, the FeCO_3_ crystals constituting the film were found as microfaceted cylinders with trigonal-pyramidal caps closely packed in random orientations^13^ (Figure S2b).

### Dissolution Evaluation at the Interface Steel–Corrosion Film

**Figure 1:**
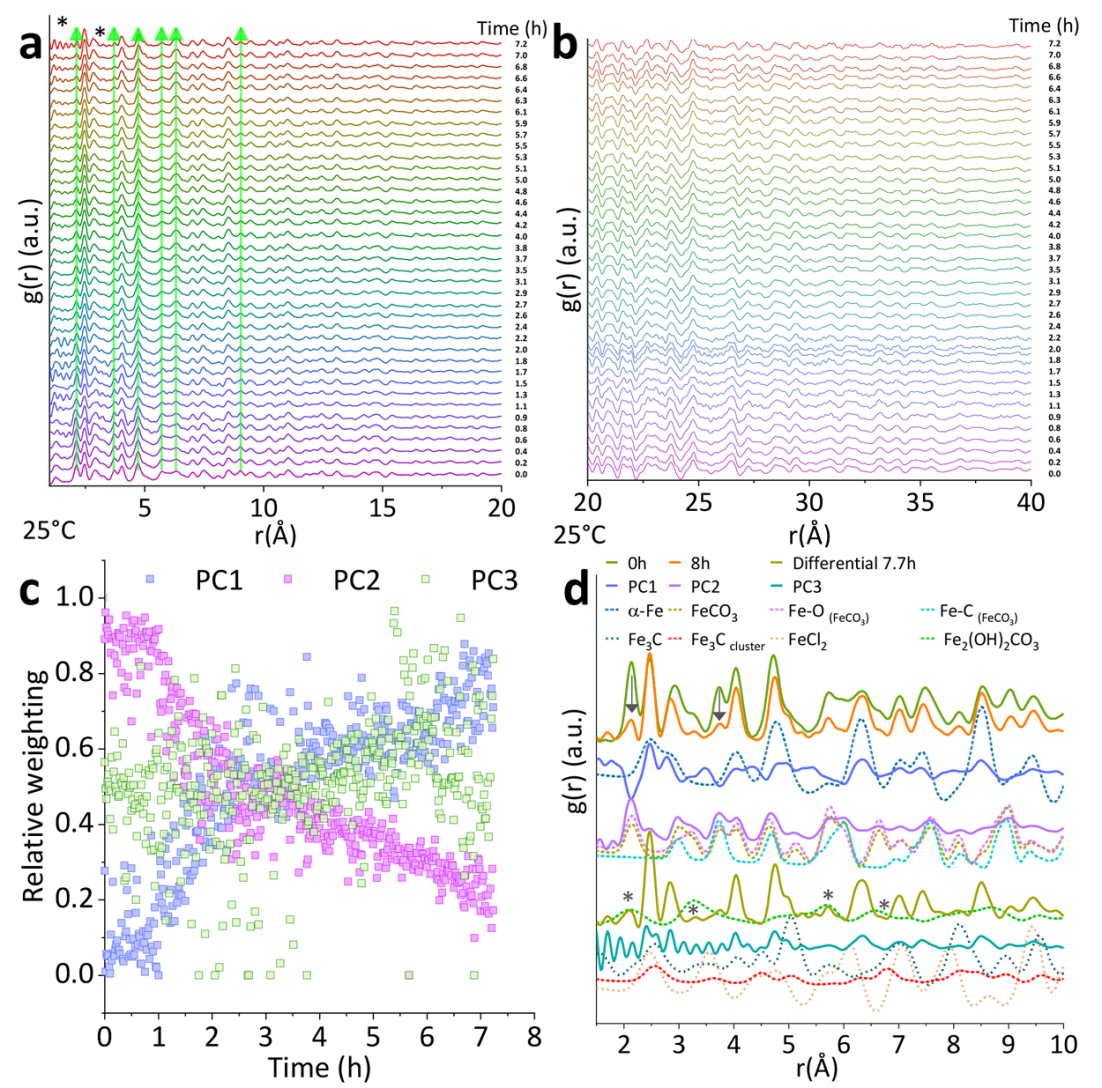
(a,b) Extracted PDFs at room temperature. (c) Principal components calculated from the extracted PDFs over the time of the reaction. (d) PDFs at the beginning *t* = 0 and at the end *t* = 7.7 h of the reaction, the differential PDF, PDFs of the principal components PC1, PC2, and PC3, and theoretical PDFs of the structures of FeCO_3_, FeCl_2_, Fe_3_C, Fe_2_(OH)_2_CO_3_, α-Fe, and PDFs of the Fe–O and Fe–C correlations in FeCO_3_.

Figure 1a,b shows PDFs up to 40 (Å^–1^) at the interface between steel and the corrosion film from the dissolution experiment over the 7.7 h at room temperature. The scans were derived from the Fourier transform of the corrected normalized total scattering. The position of the peaks corresponds to all atom–atom correlations within the system, and the integrated intensity of a PDF peak is directly related to the coordination number.^33^ Peaks at 2.13, 3.72, 4.73, 5.71, and 9.02 Å commonly appear in all scans, and their relative occurrence decreased as a function of time consistent with the dissolution process (Figure 1a,b). The general decrease of peak intensity over the time and the gradual disappearance of them beyond ∼30 Å indicate a decrease in long-range order (i.e., the loss of crystallinity) is also a consequence of this dissolution process.

To identify the structures that contribute to the pair of atoms in the PDFs, we performed PCA). Three significant components (PC1, PC2, and PC3) were derived (Figure 1c,d and Figures S3 and S4). Figure 1d shows the initial (0 h) extracted PDF and that at 7.7 h and simulated PDFs for FeCO_3_ and α-Fe. The contributions of the three PC to the data series were determined using least-squares analysis (Figure S3). In PCA, the components are mathematical constructs, and their physical meaning has to be often interpreted. Under this assumption, we identified the PC1 and PC2 contributions. The major component (PC1) relates to a phase that progressively increases over the dissolution reaction reflecting atom–atom correlations of the α-Fe structure when compared to the simulated PDF (Figures 1c,d and S3). On the contrary, the second major component (PC2) relates to a phase that progressively decreases over the dissolution reaction relating to the atom–atom correlations in the simulated PDF for FeCO_3_ (Figures 1d and S3). The final minority component (PC3) has a small contribution to the data series (0.5%), and it is less straightforward to attribute to a particular phase. To aid the identification, we calculated a differential PDF derived from the linear PDF subtraction of the FeCO_3_ and α-Fe structures from the last raw PDF scan (7.7 h) at the end of the reaction (Figures 1d and S3). The peaks of the differential PDF lie at distances of atom–atom correlations attributable to subsets of distances present in structures of α-Fe, Fe_3_C, FeCl_2_, and Fe_2_(OH)_2_CO_3_ (Figure 1d). Peaks marked with asterisks (*) in the differential PDF align with peaks from the simulated PDF for chukanovite, which could be a transient minor phase from the dissolution of siderite as the pH increases from 3.3 to 3.8, but these peaks might be instead related to other short distance atomic correlations present in the system (e.g., O–Cl). In contrast, the peak at 6.32 Å in the PDFs increases as the reaction progresses (Figure 1d), which can be attributed to Fe–Fe correlations from clusters of atoms, possibly involving a structure from the Fe–Fe_3_C type as previously suggested.^13^

**Figure 2:**
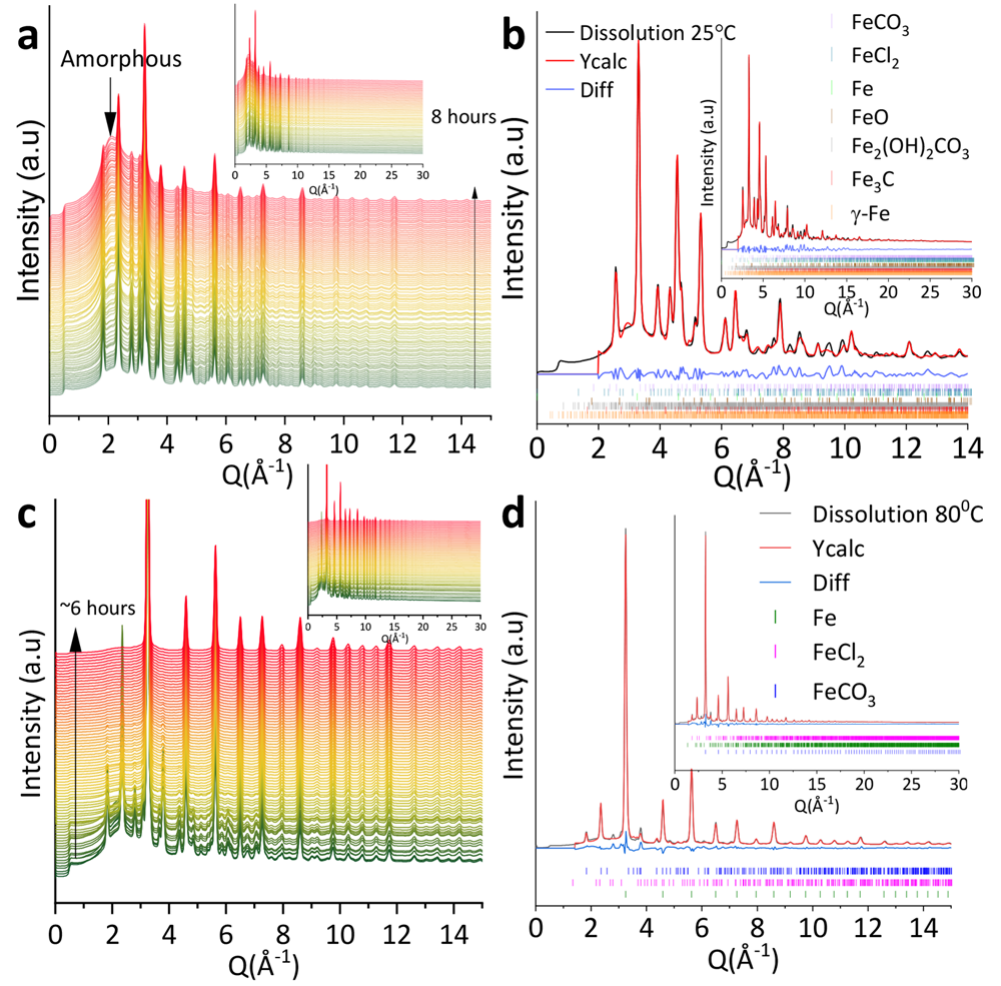
(a) Bragg data from the dissolution experiment up to 30 (Å^–1^) at room temperature and in (b) the corresponding Rietveld quantification. The fits were achieved by including FeCO_3_, α-Fe, FeCl_2_, FeO, Fe_2_(OH)_2_CO_3_, Fe_3_C, and γ-Fe and preferential orientation along planes of five of these phases. The presence of several iron phases in this complex system sharing multiple peak positions yield a quantification sensitive to preferential orientation; therefore, comparisons between data at room temperature and 80 °C are presented using a semiquantitative refinement including FeCO_3_, α-Fe, and FeCl_2_ which represents a reliable trend, as presented in Figure S5. (c) Bragg data from the dissolution experiment up to 30 (Å^–1^) at 80 °C and in (d) the Rietveld quantification for this data set.

To complement the information elucidated from the PDF data, we analyzed the Bragg portion of the data (Figure 2a). Crystalline peaks dominate the diffraction pattern series (460 scans) collected over 8 h. The evolution of a broad hump at *q* = ∼2.1 Å^–1^ indicates the formation of a poorly ordered phase material as the dissolution proceeds. We performed Rietveld phase quantification using seven iron phases with multiple peak overlap (*R*_wp_: 1.0–5.0%), which was highly sensitive to the inclusion of preferential orientation of five phases (Figure 2b). In addition, we performed a semiquantitative Rietveld refinement using three phases (FeCO_3_, FeCl_2_, and α-Fe), which although was imperfect (*R*_wp_: 2.6–10.0%), due to the complexity of the system with various phases constantly changing over time, indeed confirmed the presence of these three phases in all diffraction scans despite the overlapping peaks^34^ (Figure S5a and Table S1). The only peak of FeCO_3_ that did not overlap with peaks of the other phases present appeared at 1.8 Å^–1^ ([102̅]), and it was used to estimate a change in the integral breadth and evaluate peak shift (Figure S6a,b and Table S1). Despite the decrease in the intensity of all crystalline peaks over the time as the reaction progressed at low temperature (expected as the dissolution of FeCO_3_ indeed occurred), no signs of dissolution of the film (outer layer) were observed from imaging analysis after this experiment (Figure S7a).

Similarly, crystalline peaks dominate the diffraction pattern series (360 scans) collected from the experiment at 80 °C performed for 6.3 h (Figure 2c). Once again, the formation of an amorphous phase was observed during the first 30 min, evidenced by a broad “bump” in the diffraction pattern at ∼2.1 Å^–1^, pointing to a shorter lifetime of the amorphous phase at higher temperature. Consistently with the data at low temperature, Rietveld quantification (*R*_wp_: 4.8–6.6%) also confirmed the presence of FeCO_3_, FeCl_2_, and α-Fe over the time of the reaction. Similarly, only the peak that corresponded to the [102̅] lattice plane (at 1.8 Å^–1^) of FeCO_3_ was used to evaluate integral breadth and to estimate any shifts in the position of the peak (Figure S6b,d and Table S2).

**Figure 3:**
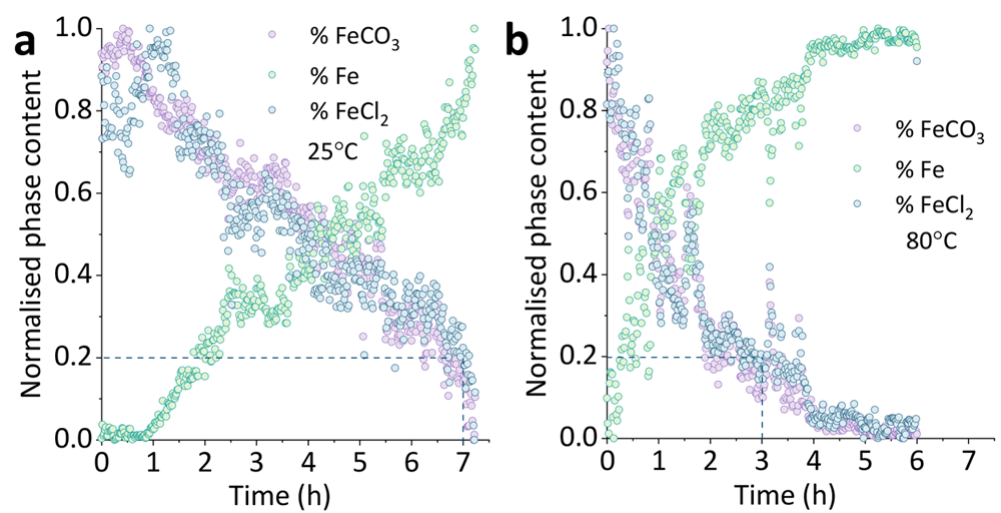
Kinetics of dissolution were for the experiment using the phase percent data extracted from Rietveld refinement: (a) at room temperature and (b) at 80 °C.

In agreement with the PDFs, Rietveld analysis showed an identical trend for the FeCO_3_ and α-Fe phases (Figure 2d). In addition, this analysis demonstrated that the relative content of FeCl_2_ simultaneously disappeared along with FeCO_3_, suggesting that FeCl_2_ evolves as an intermediate phase from the FeCO_3_ dissolution (Figure 3a,b, contribution of phases to the Bragg part of total scattering data). According to the relative content of phases over the time of the reaction, the contribution related to FeCO_3_ to the phase composition decreased by 80% within 7 h at a room temperature, while such an analogous decrease occurred at 80 °C in only 3 h and showed almost complete dissolution at 6 h (Figure S7b). This indicates a significant dissolution of the corrosion material at the interface between the corrosion film and steel that is not revealed on the film surface.

**Figure 4:**
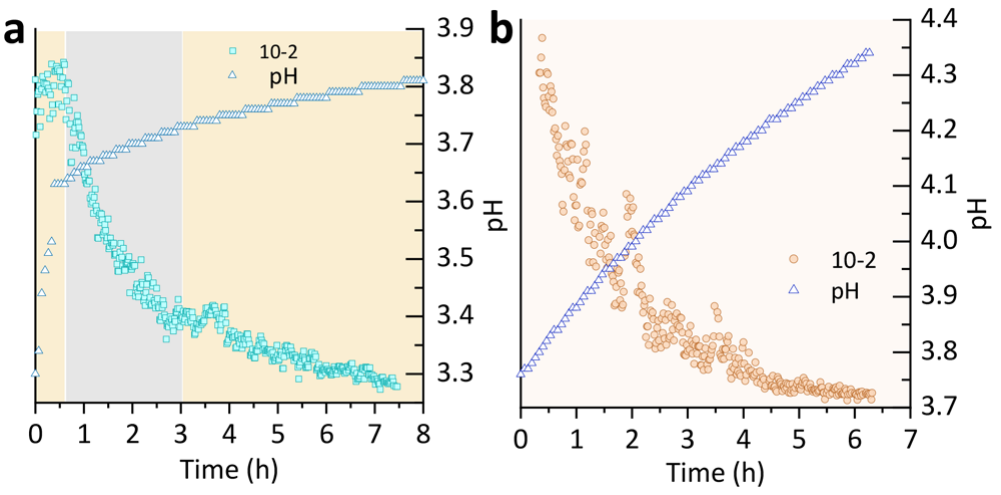
Kinetic information was also derived from the disappearance of the [102̅] lattice plane of the FeCO_3_ crystals: (a) room temperature and (b) at 80 °C through the quantification of the changes in intensity to the corresponding peak as a function of time.

Kinetics of dissolution were calculated to be 0.11% FeCO_3_·h^–1^ following a zero-order reaction for the experiment at the room temperature using the phase percentage data extracted from Rietveld quantification and presented in Figure S8a. As calculated, the kinetic rates represent the decreasing contribution of FeCO_3_ to the Bragg data accounting for all the peaks that can be attributed to this phase in the fit (Figure 2b). The FeCO_3_ dissolution rate at 80 °C was found to be two times (2×) faster than the one at the room temperature, following a pseudo-first-order reaction with a value of 0.25% FeCO_3_·h^–1^ (Figure S8b). Kinetic information was also derived from the disappearance of the [102̅] reflections of the FeCO_3_ crystals through the quantification of the changes in intensity of the corresponding peak as a function of time (Figure S8c). The extent of the disappearance of this lattice was obtained by normalizing the intensity using the expression α = *I*_*t*_/*I*_max_, where *I*_*t*_ is the intensity at a given time and *I*_max_ is a maximum area of the peak at *t* = 0. Plots of normalized intensity indicated that the kinetics of dissolution of the lattice follow a pseudo-first-order kinetic model of reaction with an exponentially decreasing trend (Figure 4, lattice [102̅]). At room temperature, the full process appears to occur in three stages. In the first stage (∼40 min), the intensity fluctuates with no tendency to decrease, suggesting that dissolution of FeCO_3_ has not been initiated; during the second stage between 40 min and ∼3 h, the [102̅] crystal lattice planes rapidly dissolved and then the process slowed down during the third stage after 3 h until the end of the experiment, overall dissolving at ∼0.20 h^–1^ (Figure 4a).

Interestingly, even if the system started with a low pH of 3.3 (to induce dissolution), the chemical dissolution of FeCO_3_ started when the pH reached 3.6, which is the pH at the equilibrium with CO_2_ injection. It is worth mentioning that, despite the general trend for the intensities to decrease within the third stage of dissolution (∼4–7.7 h), the intensity values during this stage fluctuated as the pH reached 3.8. At 80 °C, the [102̅] crystalline plane dissolved at 0.27 h^–1^, which was 1.37 times faster than the same process at room temperature (Figure 4b).

As mentioned above, despite Rietveld quantification being incomplete for some data sets in this complex time-dependent system (*R*_wp_: 9.0–10.0%), the overall trends in the data series can be useful. We used data from the refinement to give us an idea about the changes in the strain of the FeCO_3_ unit cell as a function of time (Figure S9a,b). Compression was calculated along the lattice directions *a* and *c* of the unit cell. This compression increased steadily over the 8 h of the dissolution period, and it was significantly higher during the first 2.5 h for both lattice parameters when the pH reached 3.73. However, overall lattice parameter *a* showed compression higher than that of the parameter *c*. Conversely, parameter *b*, which is parallel to the [102̅] lattice plane, showed a steady relaxation during the first 2.5 h. This was also evident from the shifts observed in the position of the peak throughout the reaction (Figure S6c). Consequently, the volume of the unit cell was affected with a steady reduction over the 8 h of the experiment (Figure S9a). Interestingly, a steady decrease in the volume of the unit cell of the α-Fe structure demonstrated that both steel and corrosion film are affected simultaneously during the dissolution processes at the interface (Figure S9c,d).

In the system at 80 °C, compression was also evidenced along the lattice parameters *a* and *c* with a steady increase during the first 2.6 h when the pH reached 4.05. For the lattice parameter *b*, however, data exhibited noise after 2.5 h, indicating extensive changes in the structure (i.e., extensive dissolution) that coincided with the major changes in the volume of the FeCO_3_ unit cell. These results agree with the findings from the experiment at low temperature in which the major strain was identified in the lattice parameter *b*, parallel to the [102̅] lattice plane. It agreed with the increase in integral breadth (broadening) quantified over the first 5 h of the reaction. The increase continued until the disappearance of the diffraction peak, explaining the large dispersion in the data (Figure S9b). Similarly, in the experiment at low temperature, the position of the peak that corresponded to the [102̅] lattice plane fluctuated over the time of dissolution possibly linked to the strains developing in the crystals as the dissolution continues.

The corrosion film on carbon steel under CO_2_ saturation conditions was identified to contain siderite FeCO_3_ with a rhombohedral crystal structure, as was previously reported in other works.^35,36^ Our study focuses strictly on the interface zone between steel and corrosion film and shows that the dissolution of the as-formed FeCO_3_ in a brine solution composed of 1% HCl involves the intermediate formation of FeCl_2_ or FeCl^+^ and entails the occurrence of an amorphous (likely γ-Fe and Fe_3_C clusters) phase. Previously, it has been suggested that FeCl^+^ is a precursor phase originating from a homogeneous distribution of Cl^–^ at the interface analyzed on cross sections by SEM during a dissolution process.^19^ In addition, a mixture of subsets of interatomic distances from the structures for α-Fe, Fe_3_C, and FeCl_2_ was quantified. These subsets of interatomic distances, including Fe_3_C and α-Fe, provide evidence for the structural damage at the interface in the beginning of the reaction. This is an indication of the damage caused at the interface, to include the thin corrosion layer, reported to be present between the thick corrosion layer (siderite) and steel.^8−10^ In other words, it is a damage to the foundations that sustain the thick corrosion film likely aided by the greater porosity of Fe_3_C that reduces the strength of this material. Furthermore, an amorphous phase was found to be present. We attempted to discover its nature by simulating the background fitted as a part of the Rietveld refinement under the assumption that this background is, in fact, an amorphous component. In the course of this fitting, the parameters of the background Chebyshev polynomial function were generated in Topas.^36−42^ The parameters were used to generate simulated background curves for the angular range and resolution identical to those used in the actual measurements. We applied this procedure to the last 14 diffraction patterns of the experiment where the amorphous phase showed higher intensity (Figure S10a), and in which the refinement quality was the best (*R*_wp_: 1.3–1.8%). From this, we found that the background curves exhibited a systematic a consistent evolution in line with an expected development of an amorphous phase. Thus, the PDFs were calculated for all 14 background curves as if they were stand-alone measurements. The PDFs obtained from the simulated background were compared against various possible phases of crystal structures or clusters of FeCO_3_, FeCl, and α-Fe; however, none of them matched. A mixture of γ-Fe and clusters of Fe_3_C aligned better with the PDFs (Figure S10b), although γ-Fe is only stable at temperatures between 915 and 1395 °C.^42^ γ-Fe is the precursor of α-Fe and Fe_3_C which form within the boundaries of austenite parental grains. However, it is known that the retained austenite can be present after a quenching process or through stabilization achieved in the presence of impurities.^43,44^ The role of retained γ-Fe is still debated, but it has been suggested to be linked to fatigue crack initiation; however, other authors report benefits,^45,46^ regardless, small but continuously increasing presence of amorphous γ-Fe and Fe_3_C is yet another indication of steel substrate degradation.

Overall, our results show that favorable conditions for dissolution FeCO_3_ also affect the integrity of steel regardless of the temperature of the reaction. Overall, kinetics of FeCO_3_ dissolution at 80 °C were found to be 2.5 times faster than the same process at low temperature; however, differential dissolution on crystalline planes were identified. For example, the [102̅] lattice plane was largely affected during the dissolution with the corresponding diffraction peak disappearing almost entirely over the relatively short duration of the experiment. This observation was further confirmed with the larger strain quantified for the lattice parameter *b*, likely because the [102̅] lattice plane coincides with the Fe atoms of the unit cell with a nonstoichiometric termination, which can be specifically targeted to be protected by inhibitor molecules (Figure S11). Our results are in good agreement with previous results that established a removal of the constituents’ ionic species perpendicular to the 104 surface.^47^ Furthermore, our results show the evolution of texture according to the change in preferential orientation along the 104 plane from limited at the beginning of the reaction to moderate at the end (0.7–0.6; a value of 1.0 corresponds to no preferred orientation), suggesting also an increase in porosity and the consequent change in the properties of the corrosion film (Figure S12a,b).

The protective nature of FeCO_3_ is recurrently mentioned in the literature, where this statement is based on notion that this compound is formed in a form of a dense and compact crystalline corrosion film, thus impeding the transport of chemical species from the solution to the steel.^11,17,20,35,48,49^ Nonetheless, the integrity of all materials at the interface can still be compromised when favorable conditions for the dissolution of the corrosion film prevail (e.g., undersaturation and pH decrease). Even if the corrosion film does not show visible damage, dissolution and mechanical strain on the unit cell translates into volume change, evolution of texture, and changes in corrosion properties reflected at the macroscale (Figures S9 and S12).

Furthermore, damaging effects of using acid solutions in cleaning operations of steel pipelines under a relatively low flow (1 m/s), as was used in this work, evidenced that besides FeCO_3_ dissolution pit formation also occurred. This damage was captured by the interferometry analysis at the end of the experiment at 80 °C. Figure S13 (box plots in the SI) shows the irregularity of the surface with 50% of the pits being between 25 and 50 μm in diameter and 13–18 μm in depth; however, few pits between 100 and 250 μm in depth were quantified, demonstrating the major damage to the steel substrate (Figure S14), a depth that not a single layer of corrosion film can amend.

## Conclusions

Our work on the dissolution of corrosion films in sweet environments, under acidic and undersaturated conditions, demonstrated that the effects in the integrity of steel is far more significant than the damage observed on the surface of the corrosion film. By identifying and quantifying the changes in the corrosion products at the interface, between corrosion film and steel, we proved that dissolution of FeCO_3_ involves the presence of an amorphous phase, the intermediate formation of FeCl_2_ or FeCl^+^, and the presence of a phase with short distance atom–atom correlations. The amorphous phase was identified as a mixture of retained γ-Fe and Fe3C.

In addition, damage of material at the interface (thin layer between the thick corrosion film and steel) evidenced the presence of clusters of atoms from α-Fe and Fe_3_C structures, demonstrating the structural damage of these phases at a molecular level and, therefore, damage to the foundations that support the thick corrosion film. This is, in fact, not reflected on the film surface having almost an intact appearance. This proves the benefit of studying corrosion reactions at the microscale to reveal information that is not reflected on the corrosion film surface.

In order to deepen our knowledge on the dissolution of FeCO_3_, we quantified a differential dissolution of crystalline lattices of the crystalline structure of siderite, where it is the [102̅] lattice plane that dissolves more rapidly. The fast dissolution nature of this lattice is likely due to the nonstoichiometric termination of Fe atoms. This information can be used to develop a protection strategy aimed at using inhibitor molecules that protect the [102̅] lattice plane. Our results also demonstrate that not only the unit cell of FeCO_3_ is affected but also that of α-Fe from steel, even at low temperature. This was quantified with pit depths of up to 250 μm. Our work proves that studying exclusively corrosion films is not sufficient to evaluate the condition of steel, and further research is needed regarding the interactive zone between steel and corrosion film. As we demonstrated, the consequences are far more serious than previously thought using relatively mild conditions for the dissolution of corrosion film. The impact on the integrity of steel can be, therefore, more drastic than frequently reported in industrial operations of CO_2_ transport industries that use cleaning procedures (e.g., acid treatment, pigging) as part of their operational activities.

## Supporting Information Available

Figures showing the experimental setup for synchrotron experiments on I15-1 at diamond light source; characterization on initial corrosion film (diffraction and SEM image) before and after dissolution; diffraction peaks identified after dissolution of corrosion film at room temperature and 80 °C using 1% NaCl and pH ∼3.6 for ∼7–8 h; dissolution kinetics calculated from the % FeCO_3_ contributing to the Bragg part of the scattering data obtained from Rietveld refinement; PDFs extracted from total scattering data collected at the interface between corrosion films and steel during a dissolution reaction; eigenvalues from PCA for the dissolution experiments; PFD extracted from PCA analysis from dissolution experiment at low temperature; background simulation using the background parameters modeled in the Rietveld refinement using the Chebyshev polynomial function in Topas; a 2 × 2 × 2 until cell of FeCO_3_ showing the [102̅] crystal lattice illustrating the Fe atoms of the unit cell with a nonstoichiometric termination; preferred orientation along the dissolution process at room temperature and 80 °C; pit mapping after dissolution at 80 °C; tables showing the diffraction peaks of the three distinct phases identified on the patterns after dissolution at room temperature and 80 °C (PDF)

## References

[ref1] Bell E.; Cullen J. United Nations/Framework Convention on Climate Change Adoption of the Paris Agreement, 21st Conference of the Parties; United Nations: Paris, 2015.

[ref2] Franssen J. M.; Vila Real P. Annex C: Mechanical Properties of Carbon Steel and Stainless Steel. Wiley Online 2013, 359–381. 10.1002/9783433601570.app3.

[ref3] Nordsveen M.; Nesic S.; Nyborg R.; Stangeland A. A mechanistic model for carbon dioxide corrosion of mild steel in the presence of protective iron carbonate films- Part 1: Theory and Verification. Corrosion 2003, 59, 443–456. 10.5006/1.3277576.

[ref4] Choi Y.-S.; Nešić S. Determining the corrosive potential of CO2 transport pipeline in high pCO2–water environments. Int. J. Greenh. Gas Control 2011, 5, 788–797. 10.1016/j.ijggc.2010.11.008.

[ref5] Dugstad A.; Halseid M.; Morland B. Effect of SO2 and NO2 on Corrosion and Solid Formation in Dense Phase CO2 Pipelines. Energy Procedia 2013, 37, 2877–2887. 10.1016/j.egypro.2013.06.173.

[ref6] Boot-Handford M. E.; Abanades J. C.; Anthony E. J.; Blunt M. J.; Brandani S.; Mac Dowell N.; Fernández J. R.; Ferrari M.-C.; Gross R.; Hallett J. P. J. E.; et al. Carbon capture and storage update. Energy Environ. Sci. 2014, 7, 130–189. 10.1039/C3EE42350F.

[ref7] Popescu C.; Gabor M. R. Quantitative Analysis Regarding the Incidents to the Pipelines of Petroleum Products for an Efficient Use of the Specific Transportation Infrastructure. Processes 2021, 9, 1535 10.3390/pr9091535.

[ref8] Gulbrandsen E.; Morard J. H. Study of the Possible Mechanisms of Steel Passivation in CO_2_ Corrosion; NACE International, 1999.

[ref9] Han J.; Nešić S.; Yang Y.; Brown B. N. Spontaneous passivation observations during scale formation on mild steel in CO2 brines. Electrochim. Acta 2011, 56, 5396–5404. 10.1016/j.electacta.2011.03.053.

[ref10] Bajt Leban M.; Kosec T. Characterization of corrosion products formed on mild steel in deoxygenated water by Raman spectroscopy and energy dispersive X-ray spectrometry. Eng. Fail. Anal. 2017, 79, 940–950. 10.1016/j.engfailanal.2017.03.022.

[ref11] Farelas F.; Brown B.; Nesic S. Iron Carbide and its Influence on the Formation of Protective Iron Carbonate in CO_2_ Corrosion of Mild Steel; NACE International, 2013; pp 1–15.

[ref12] López D. A.; Schreiner W. H.; de Sánchez S. R.; Simison S. N. The influence of carbon steel microstructure on corrosion layers: An XPS and SEM characterization. Appl. Surf. Sci. 2003, 207, 69–85. 10.1016/S0169-4332(02)01218-7.

[ref13] Matamoros-Veloza A.; Barker R.; Vargas S.; Neville A. Mechanistic Insights of Dissolution and Mechanical Breakdown of FeCO3 Corrosion Films. ACS Appl. Mater. & Interfaces 2021, 13, 5741–5751. 10.1021/acsami.0c18976. 33475361

[ref14] Farelas F.; Galicia M.; Brown B.; Nesic S.; Castaneda H. Evolution of dissolution processes at the interface of carbon steel corroding in a CO2 environment studied by EIS. Corros. Sci. 2010, 52, 509–517. 10.1016/j.corsci.2009.10.007.

[ref15] Staicopolus D. N. The Role of Cementite in the Acidic Corrosion of Steel. Journal of J. Electrochem. Soc. 1963, 110 (11), 1121–1124. 10.1149/1.2425602.

[ref16] Videm K. The influence of the state of the surface on the electrochemistry of iron and carbon steel electrodes in aqueous CO2 solutions. Surf. Sci. 1995, 335, 235–240. 10.1016/0039-6028(95)00422-X.

[ref17] Dugstad A. Mechanism of Protective Film Formation During CO2 Corrosion of Carbon Steel; NACE International, 1998; pp 1–11.

[ref18] Berntsen T.; Seiersten M.; Hemmingsen T. Effect of FeCO3 Supersaturation and Carbide Exposure on the CO2 Corrosion Rate of Carbon Steel. Corrosion 2013, 69, 601–613. 10.5006/0553.

[ref19] Mundhenk N.; Carrero S.; Knauss K. G.; Wonneberger R.; Undisz A.; Wu Y. Kinetic and thermodynamic analysis of high-temperature CO2 corrosion of carbon steel in simulated geothermal NaCl fluids. Corros. Sci. 2020, 171, 108597 10.1016/j.corsci.2020.108597.

[ref20] Crolet J. L.; Thevenot N.; Nešić S. J. C. Role of Conductive Corrosion Products in the Protectiveness of Corrosion Layers; NACE International, 1998; pp 194–203.

[ref21] Al-Hassan S.; Mishra B.; Olson D. L.; Salama M. M. Effect of Microstructure on Corrosion of Steels in Aqueous Solutions Containing Carbon Dioxide. Corrosion 1998, 54, 480 10.5006/1.3284876.

[ref22] Kermani M. B.; Morshed A. Carbon Dioxide Corrosion in Oil and Gas Production—A Compendium. Corrosion 2003, 59, 659–683. 10.5006/1.3277596.

[ref23] Pessu F.; Barker R.; Neville A. Pitting and Uniform Corrosion of X65 Carbon Steel in Sour Corrosion Environments: The Influence of CO_2_, H_2_S, and Temperature. Corrosion 2017, 73, 1168–1183. 10.5006/2454.

[ref24] Sun W.; Li C.; Ling S.; Reddy R. V.; Pacheco J. L.; Asmann M.; Wilken G.; Franco R. J. Laboratory Study of Sour Localized/Pitting Corrosion. Presented at the CORROSION 2011, Houston, TX, 2011.

[ref25] Effenberger H.; Mereiter K.; Zemann J. Crystal structure refinements of magnesite, calcite, rhodochrosite, siderite, smithonite, and dolomite, with discussion of some aspects of the stereochemistry of calcite type carbonates. Zeitschrift fur Kristallographie 1981, 156, 233–244. 10.1524/zkri.1981.156.14.233.

[ref26] Filik J.; Ashton A. W.; Chang P. C. Y.; Chater P. A.; Day S. J.; Drakopoulos M.; Gerring M. W.; Hart M. L.; Magdysyuk O. V.; Michalik S.; Smith A.; Tang C. C.; Terrill N. J.; Wharmby M. T.; Wilhelm H. Processing two-dimensional X-ray diffraction and small-angle scattering data in DAWN 2. J. Appl. Crystallogr. 2017, 50, 959–966. 10.1107/S1600576717004708. 28656043 PMC5458597

[ref27] Soper A. K. GudrunN and GudrunX: Programs for Correcting Raw Neutron and X-ray Diffraction Data to Differential Scattering Cross Section. Tech. Rep. RAL-TR-2011-013; Rutherford Appleton Laboratory, 2011.

[ref28] Soper A. K.; Barney E. R. Extracting the pair distribution function from white-beam X-ray total scattering data. J. Appl. Crystallogr. 2011, 44, 714–726. 10.1107/S0021889811021455.

[ref29] Origin Pro; OriginLab: Northampton, MA, 2019.

[ref30] Chapman K. W.; Lapidus S. H.; Chupas P. J. Applications of principal component analysis to pair distribution function data. J. Appl. Crystallogr. 2015, 48 (6), 1619–1626. 10.1107/S1600576715016532.

[ref31] Farrow C. L.; Juhas P.; Liu J. W.; Bryndin D.; Božin E. S.; Bloch J.; Proffen T.; Billinge S. J. L. PDFfit2 and PDFgui: computer programs for studying nanostructure in crystals. J. Condens. Matter Phys. 2007, 19, 335219 10.1088/0953-8984/19/33/335219. 21694142

[ref32] Evans J. S. O. Advanced Input Files & Parametric Quantitative Analysis Using Topas. Mater. Sci. Forum 2010, 651, 1–9. 10.4028/www.scientific.net/MSF.651.1.

[ref33] aEgami T.; Billinge S. J. Underneath the Bragg peaks: Structural Analysis of Complex Materials; Newnes, 2012.

[ref34] Joshi G. R.; Cooper K.; Zhong X.; Cook A. B.; Ahmad E. A.; Harrison N. M.; Engelberg D. L.; Lindsay R. Temporal evolution of sweet oilfield corrosion scale: Phases, morphologies, habits, and protection. Corros. Sci. 2018, 142, 110–118. 10.1016/j.corsci.2018.07.009.

[ref35] Duckworth O. W.; Martin S. T. Role of molecular oxygen in the dissolution of siderite and rhodochrosite1 1Associate editor: U. Becker. Geochim. Cosmochim. Acta 2004, 68, 607–621. 10.1016/S0016-7037(03)00464-2.

[ref36] https://topas.awh.durham.ac.uk/doku.php?id=background_polynomial (accessed 2023-02-16).

[ref37] https://mathworld.wolfram.com/ChebyshevPolynomialoftheFirstKind.html (accessed on 2022-09-27).

[ref38] Oliphant T. E. Guide to NumPy; AnacondaWorks: Austin, TX, 2006.

[ref39] McKinney W. Data structures for statistical computing in python Proceedings of the 9th Python in Science Conference, Austin, TX, 2010; pp 51–56.

[ref40] https://zenodo.org/record/7223478#.Y3TEX9KZNMY (accessed on 2022-09-27).

[ref41] Juhas P.; Davis T.; Farrow C. L.; Billinge S. J. L. PDFgetX3: a rapid and highly automatable program for processing powder diffraction data into total scattering pair distribution functions. Journal of applied crystallography 2013, 46 (2), 560–566. 10.1107/S0021889813005190.

[ref42] Dossett J. L.; Boyer H. E. Fundamentals of the Heat Treating of Steel. Practical Heat Treating; ASM International, 2006.

[ref43] Fida Hassan S.; AlWadei H. Ultrahigh strength ductile microalloyed steel with a very low yield ratio developed by quenching and partitioning heat treatment. Sci. Rep. 2022, 12, 7949 10.1038/s41598-022-11722-7. 35562404 PMC9106733

[ref44] Ohaeri E. G.; Jack T.; Yadav S.; Szpunar J.; Zhang J.; Qu J. EBSD Microstructural studies on quenched-tempered API 5L X65 pipeline steel. Philos. Mag. 2021, 101, 1895–1912. 10.1080/14786435.2021.1946189.

[ref45] Sidoroff C.; Perez M.; Dierickx P.; Girodin D. Advantages and shortcomings of retained austenite in bearing steels: a review; AnacondaWorks: Austin, TX, 2014; p 1, 10.1520/STP158020140081.

[ref46] Gui X.; Gao G.; An B.; Misra R. D. K.; Bai B. Relationship between non-inclusion induced crack initiation and microstructure on fatigue behavior of bainite/martensite steel in high cycle fatigue/very high cycle (HCF/VHCF) regime. Mater. Sci. and Eng. A 2021, 803, 140692 10.1016/j.msea.2020.140692.

[ref47] Renard F.; Putnis C. V.; Montes-Hernandez G.; King H. E. Siderite dissolution coupled to iron oxyhydroxide precipitation in the presence of arsenic revealed by nanoscale imaging. Chem. Geol. 2017, 449, 123–134. 10.1016/j.chemgeo.2016.12.001.

[ref48] Ruzic V.; Veidt M.; Nesic S. Protective Iron Carbonate Films Part 1: Mechanical Removal in Single-Phase Aqueous Flow. Corrosion 2006, 62, 419 10.5006/1.3278279.

[ref49] Ruzic V.; Veidt M.; Nessic S. Protective Iron Carbonate Films Part 2: Chemical Removal by Dissolution in Single-Phase Aqueous Flow. Corrosion 2006, 62, 598–611. 10.5006/1.3280674.

