# Supplementary material for: Study of a Local Structure at the Interface between Corrosion Films and Carbon Steel Surface in Undersaturated CO2 Environments
Source: ACS Omega. 2023 Feb 23;8(9):8497–504. doi: 10.1021/acsomega.2c07631 (PMC9996759; doi:10.1021/acsomega.2c07631)
Supplement: Supplementary file 1 — ao2c07631_si_001.pdf [file ao2c07631_si_001.pdf]

Supplementary Information

The study of a local structure at the interface between corrosion films and carbon steel surface in undersaturated CO2 environments

Adriana Matamoros-Veloza*[a,b], Tomasz M. Stawski[c], Silvia Vargas[d], Anne Neville[b]

[a] Faculty of Engineering and Physical Sciences, University of Leeds, LS2 9JT, UK

[b] Institute of Functional Surfaces, School of Mechanical Engineering, University of Leeds, Leeds, LS2 9JT, UK

[c] Federal Institute for Materials Research and Testing (BAM), Richard-Willstatter-Straße 11, 12489 Berlin, Germany

[d] BP America, Inc., Houston, Texas 77079, United States

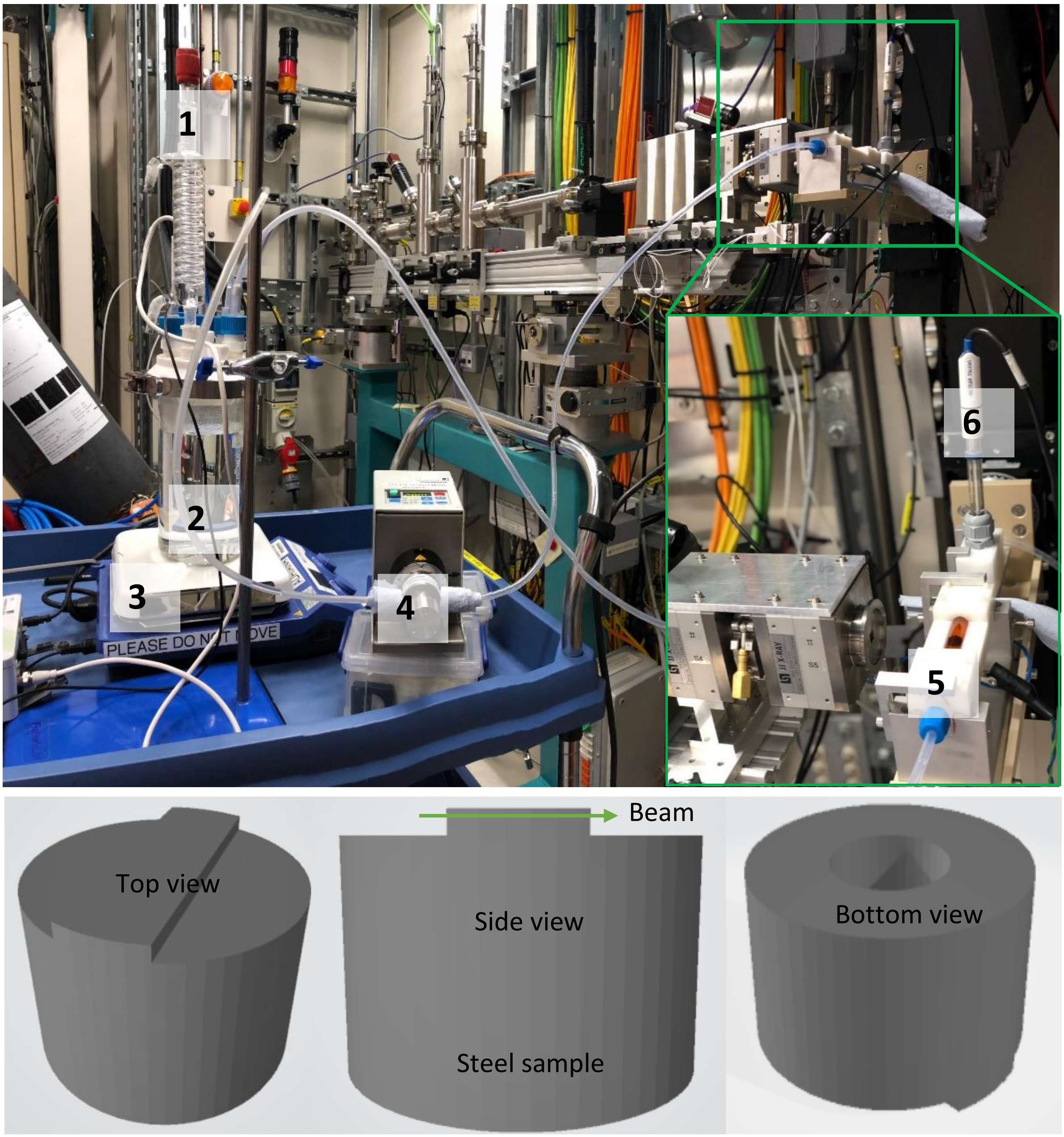

Figure S1 (**Top**) Experimental setup (1:Condenser, 2: reactor with lid; 3 hot plate; 5: pump; 5: flow-cell with a working electrode; 6: reference electrode)for the dissolution reaction at the interface between steel and corrosion scale dissolution under turbulent flow velocity of 1 m/s, 1% NaCl, pH 3.3 at room temperature and 80°C performed at the Diamond beamline I15-1. (**Bottom**) Sample with a top hat geometry with a landing step of 3 mm wide and a step height of 5mm (Bottom).

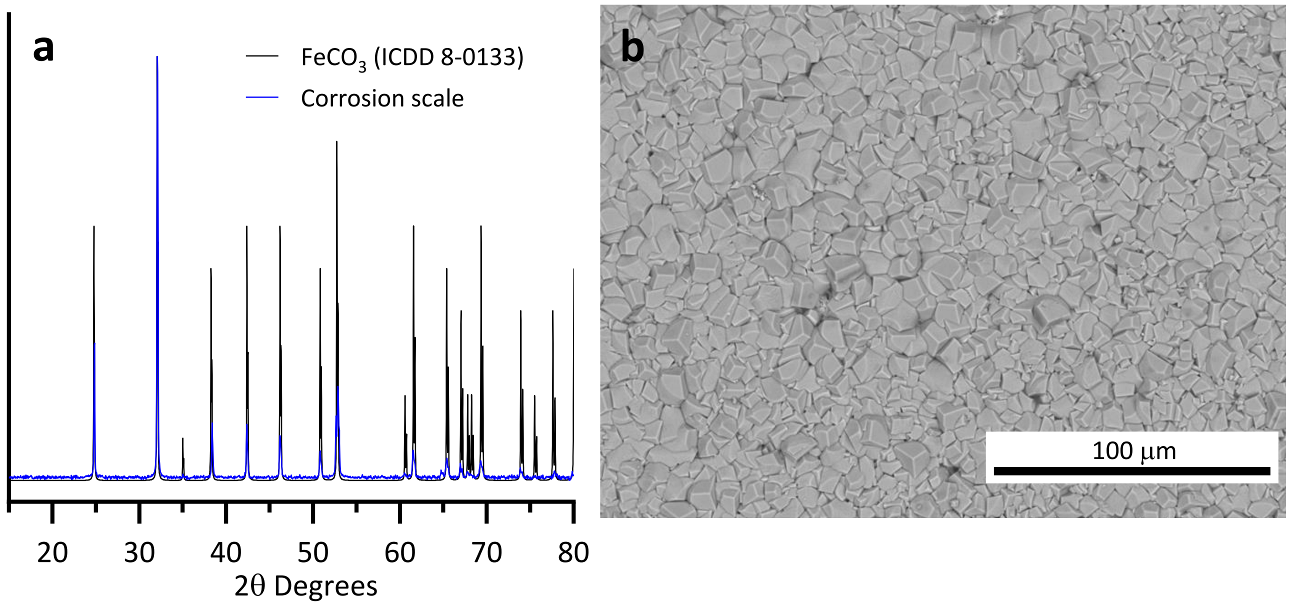

Figure S2 **a)** Diffraction pattern and **b)** SEM image of the corrosion scale at the initial state before dissolution.

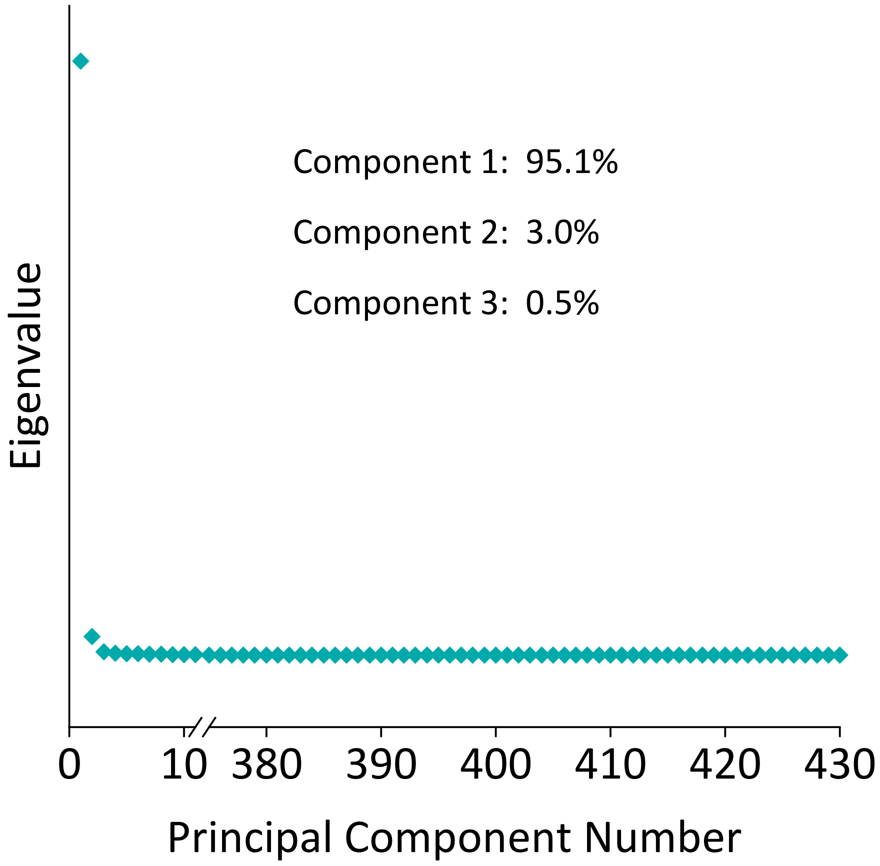

Figure S3 Eigenvalues from PCA for the dissolution experiment of 1 m/s, 1% NaCl, pH 3.3 at room temperature, and b) under the same conditions but at turbulent flow velocity of 1 m/s, 1% NaCl, pH 3.6 at 80°C.

Supplementary Note 1

Principal Component Analysis (PCA) can used as an initial step in interpreting the data without any prior knowledge of the structure or structural model (Chapman et al 2015). Significant components identified through PCA from a series of PDF data provide an indication of how PDFs (and the associated atomic structure) change over the time of the reaction, representing the original PDFs as linear combinations of these components. Each component groups features correlations associated with atom-atom distances that change over time. PDFs have peaks at *r* values that correspond to atom-atom distances within the studied material.

Relative contributions of the significant components correlate to the relative abundance of different constituents within the material. Without constraints the raw PCA output can include components and weightings that are linear combinations of PDFs and abundances for distinct phases within the sample. For the interpretation:

i) Component PDFs corresponding to the PDF for part of the sample cannot have peaks with negative intensity, which is below the ($-4\pi\rho r$)
ii) Component PDFs corresponding to the PDF for part of the sample cannot have negative weighting for that component.

To run PCA analysis the data for $r > 1$ Å were analysed because this value represents the shortest possible bond distance in the studied materials and including smaller r values is unphysical. Only 2 or 3 components are relevant which can be subtracted from the last raw PDF to get further information about the structure of the amorphous material.

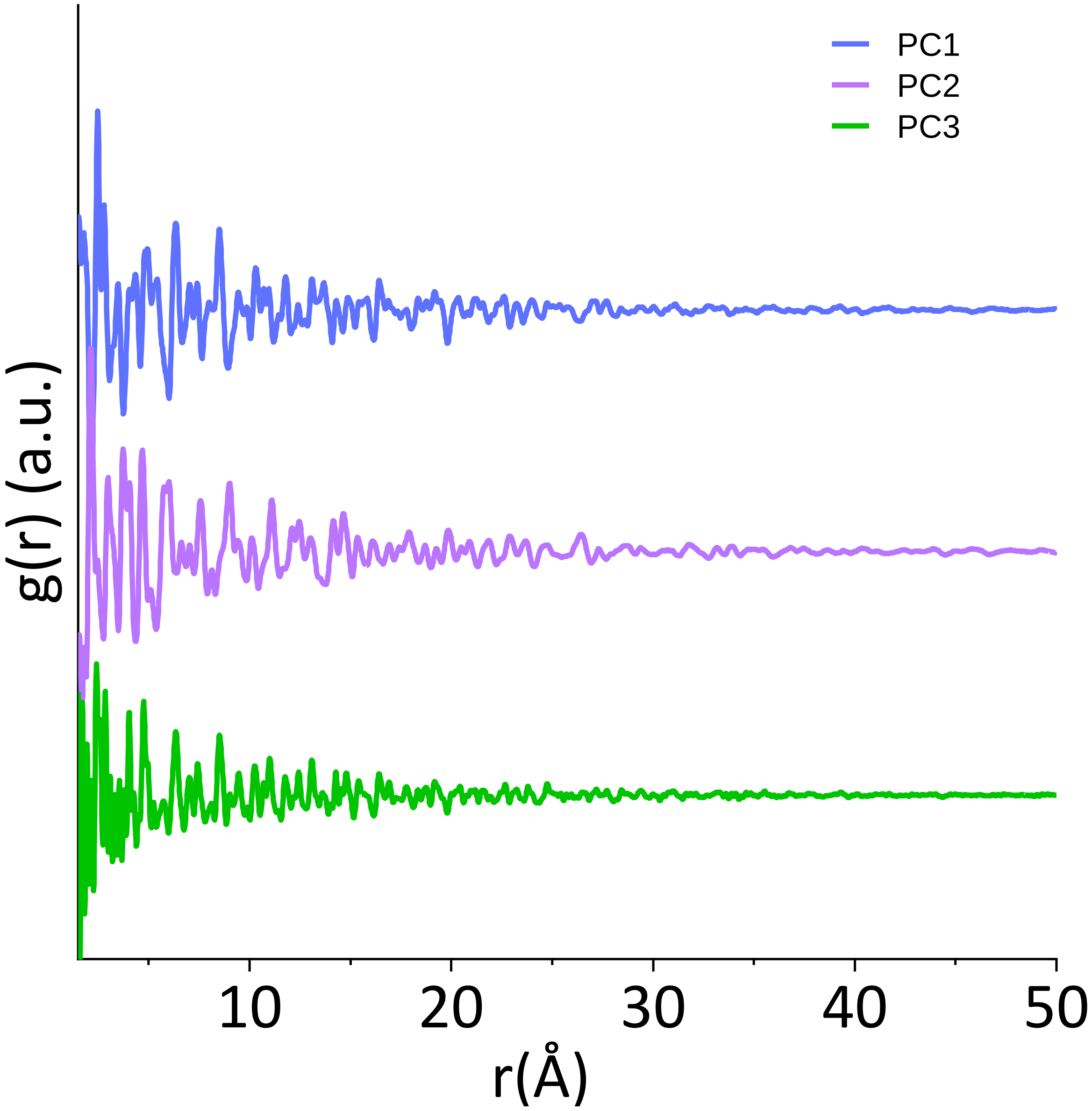

Figure S4. PDFs extracted from PCA analysis of the raw data for experiment performed under CO2 saturated conditions, 1%HCl, 1 m/s at 25°C.

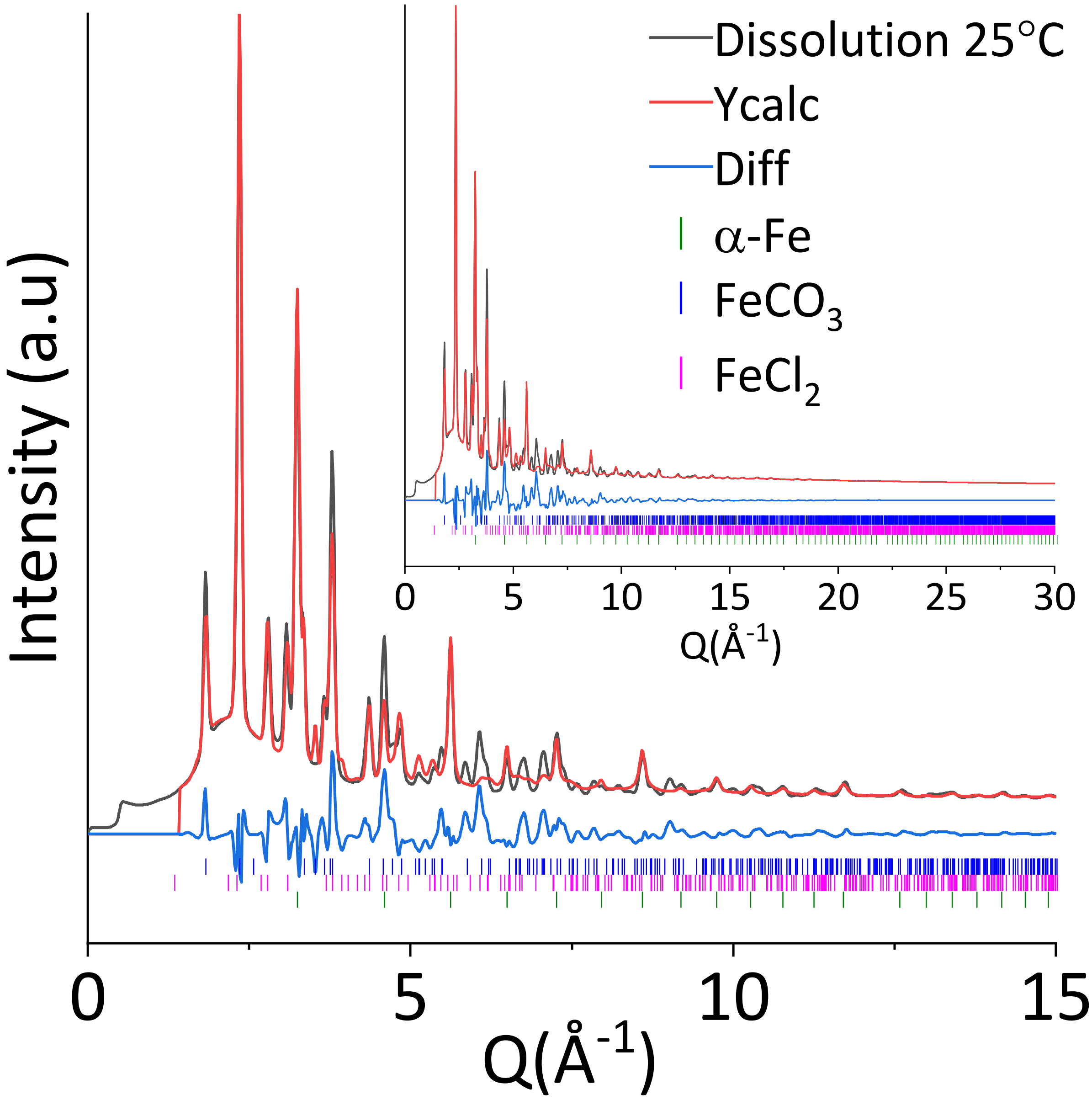

Figure S5 Semi-quantitative Rietveld refinement using $FeCO_3$, $FeCl_2$ and α-Fe that provide a good trend in the changes of the phases over time and yield comparable results in the change of the unit cell of these structures in the system.

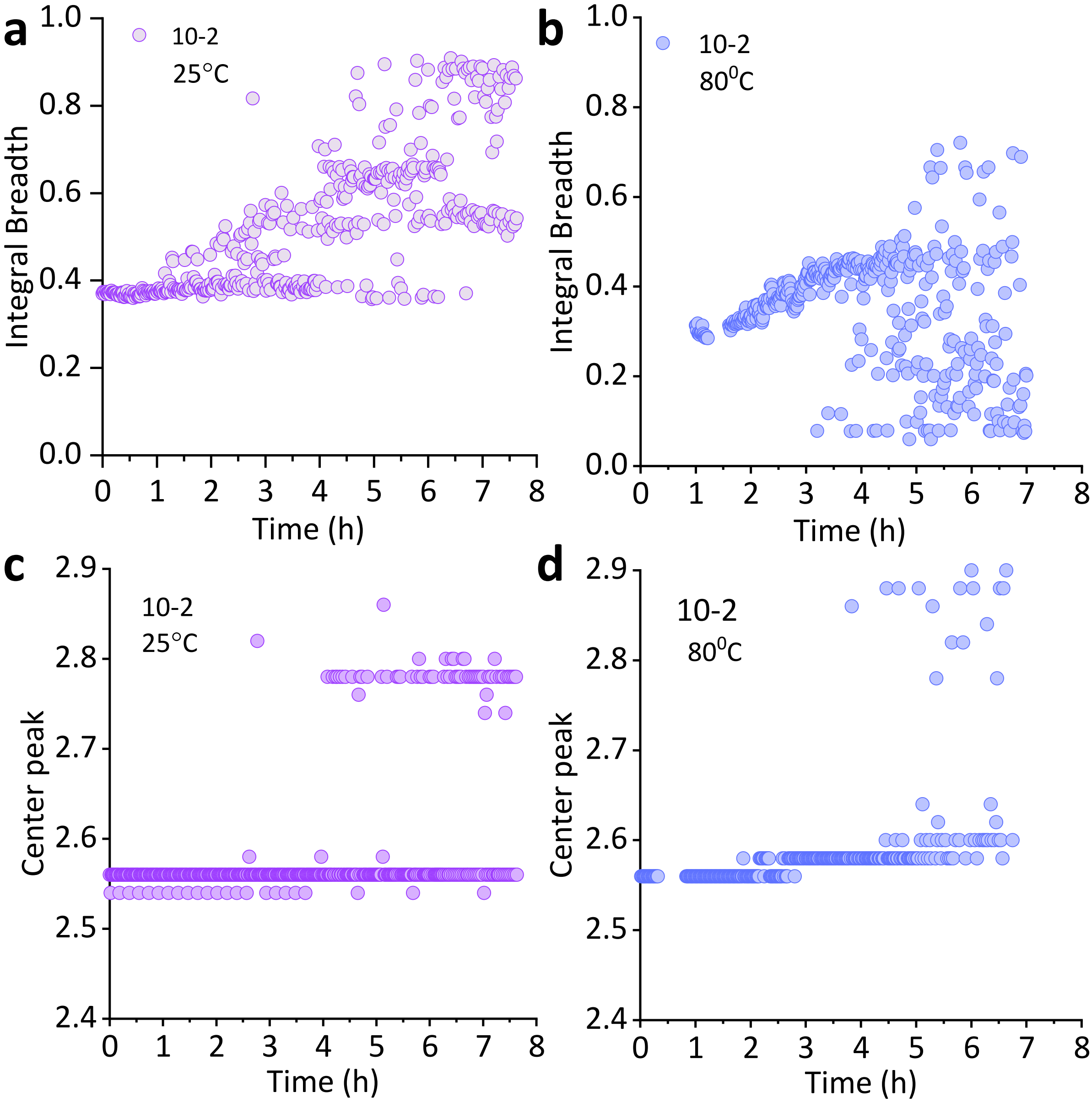

Figure S6 a) Integral breadth of the corresponding peaks for the 10-2 lattice at room temperature; **b)** same as (a) for 10-2 lattice at 80°C; **c)** peak centre position for the 10-2 lattice at room temperature; **d)** same as (c) for lattice 10-2 at 80°C.

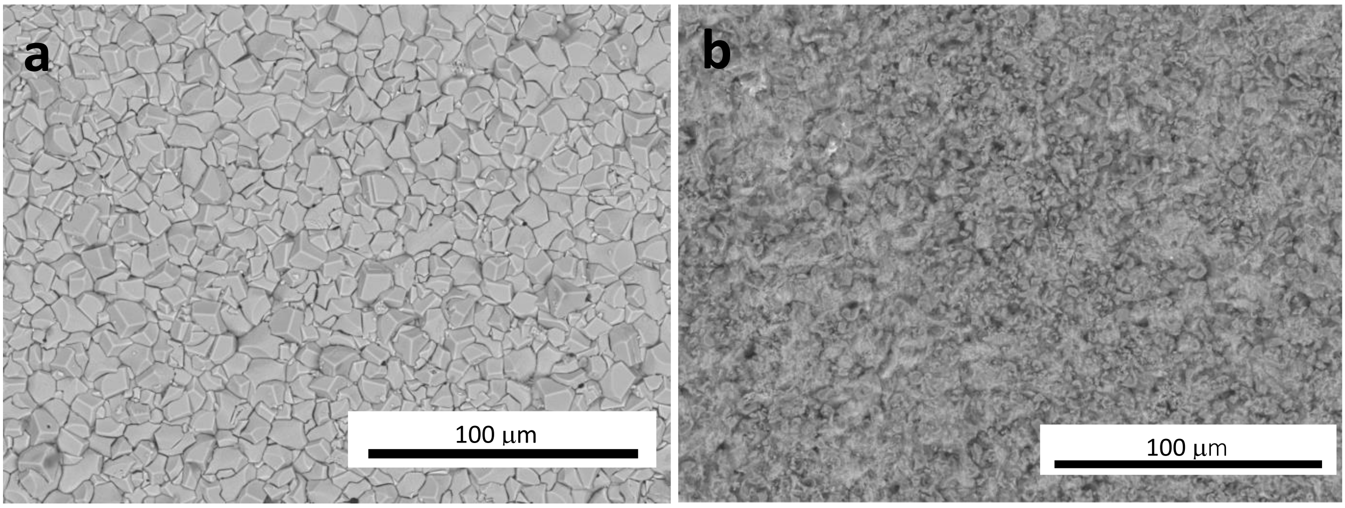

Figure S7 SEM images of **a)** corrosion scale after dissolution under turbulent flow velocity of 1 m/s, 1% NaCl, pH 3.3 at room temperature, and **b)** under the same conditions but at turbulent flow velocity of 1 m/s, 1% NaCl, pH 3.6 at 80°C.

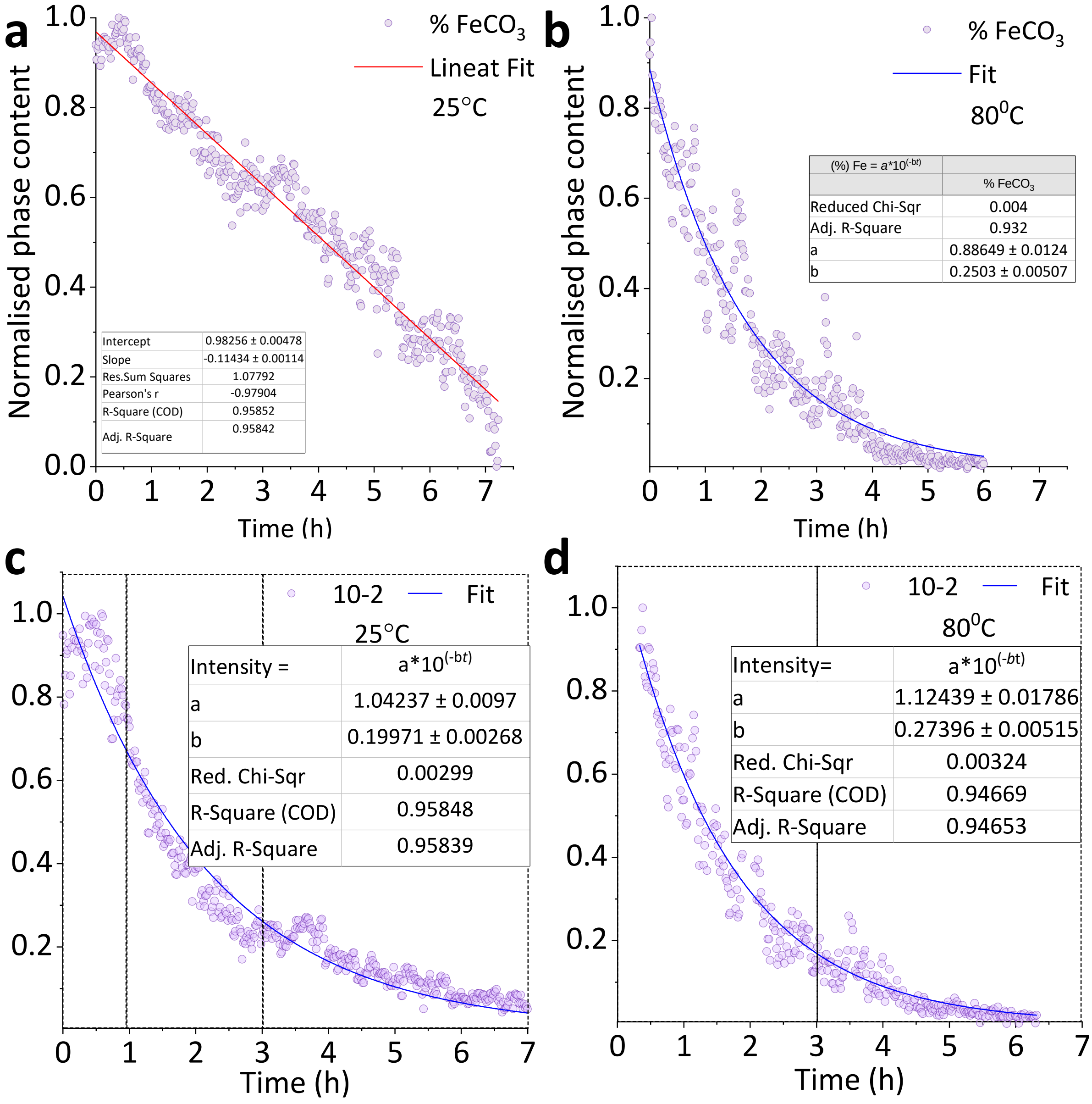

Figure S8. Kinetics of dissolution, % FeCO3 contributing to the Bragg part of the scattering data obtained from Rietveld refinement: **a)** at room temperature; **b)** at 80°C. Degree of disappearance of the 10-2 lattice obtained by normalising the intensity using the expression $\alpha = I_t/I_{max}$, where I_t is the intensity at a given time and I_{max} is a maximum area of the peak at t=0 at room temperature: **c)** Room temperature; **d)** 80°C.

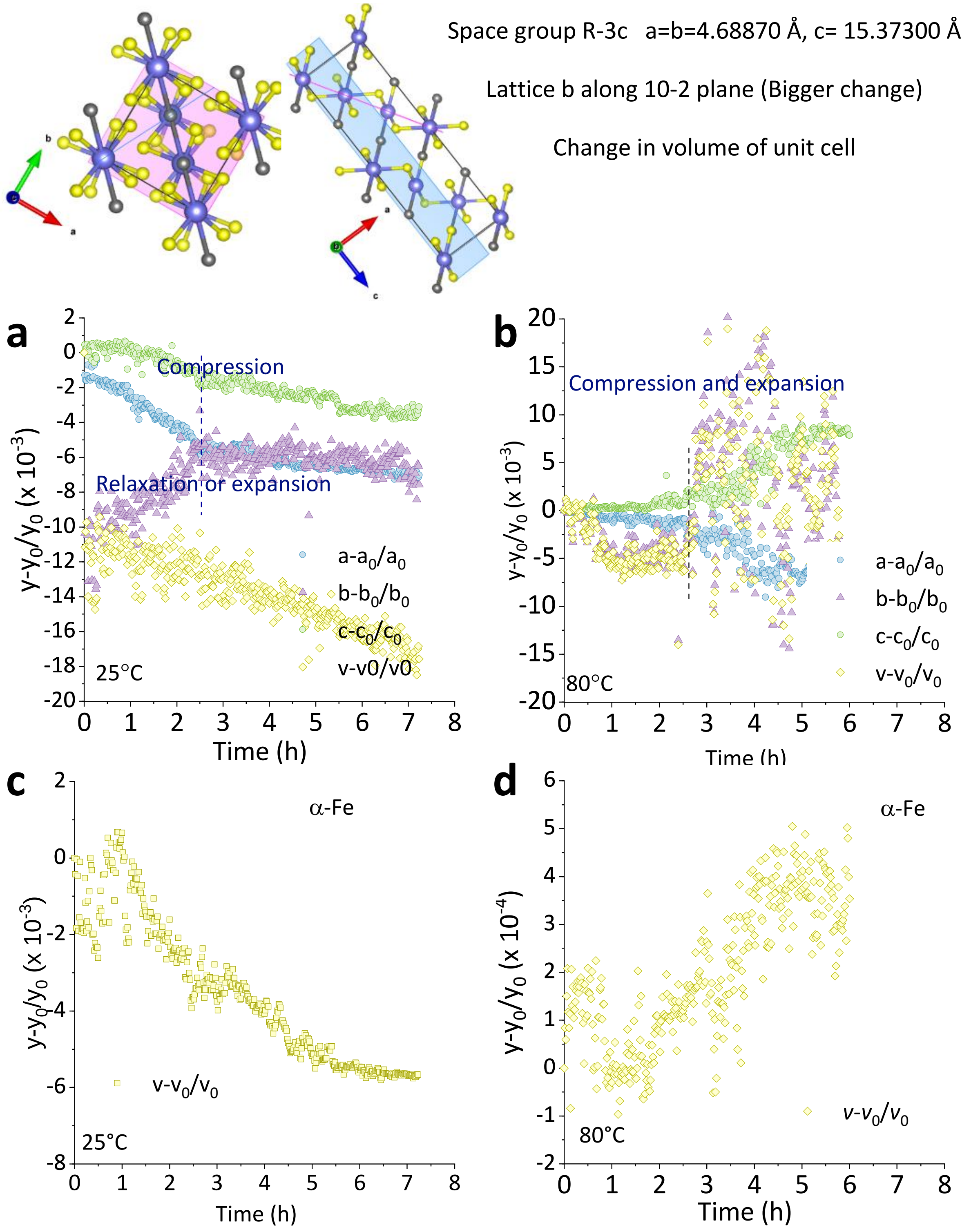

Figure S9 Strains along the a,b,c lattice parameters of the unit cell of **a)** FeCO3 at room temperature; **b)** FeCO3 at room temperature at 80°C; Unit cell volume of **c)** α-Fe at room temperature; and **d)** α-Fe at 80°C.

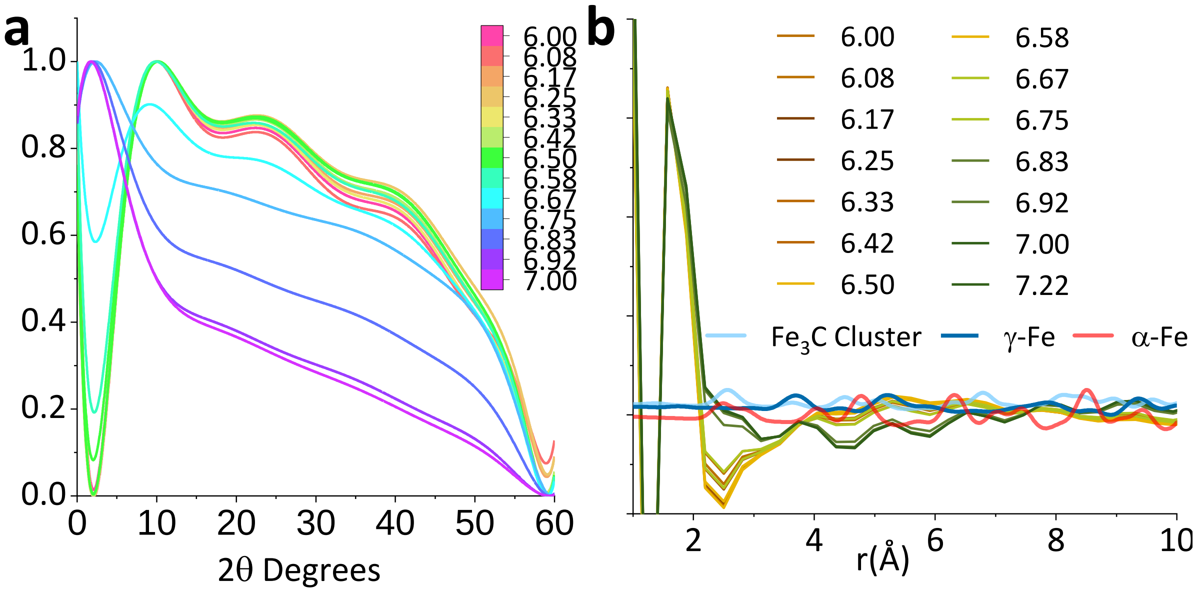

Figure S10. a) Background simulation using the background parameters modelled in the Rietveld refinement using the Chebyshev polynomial function in Topas. The simulation was obtained using a Python script and converted to PDF in pdfgetx3; We include the script as well as the data required to recreate the simulated curves; see supporting script file; **b)** PDFs extracted from simulated background curves. Worth to note that in this paper, we compared PDFs extracted from total scattering experimental data using Gudrun with simulated PDFs obtained in pdfgetx3, which both software packages yield equivalent results.

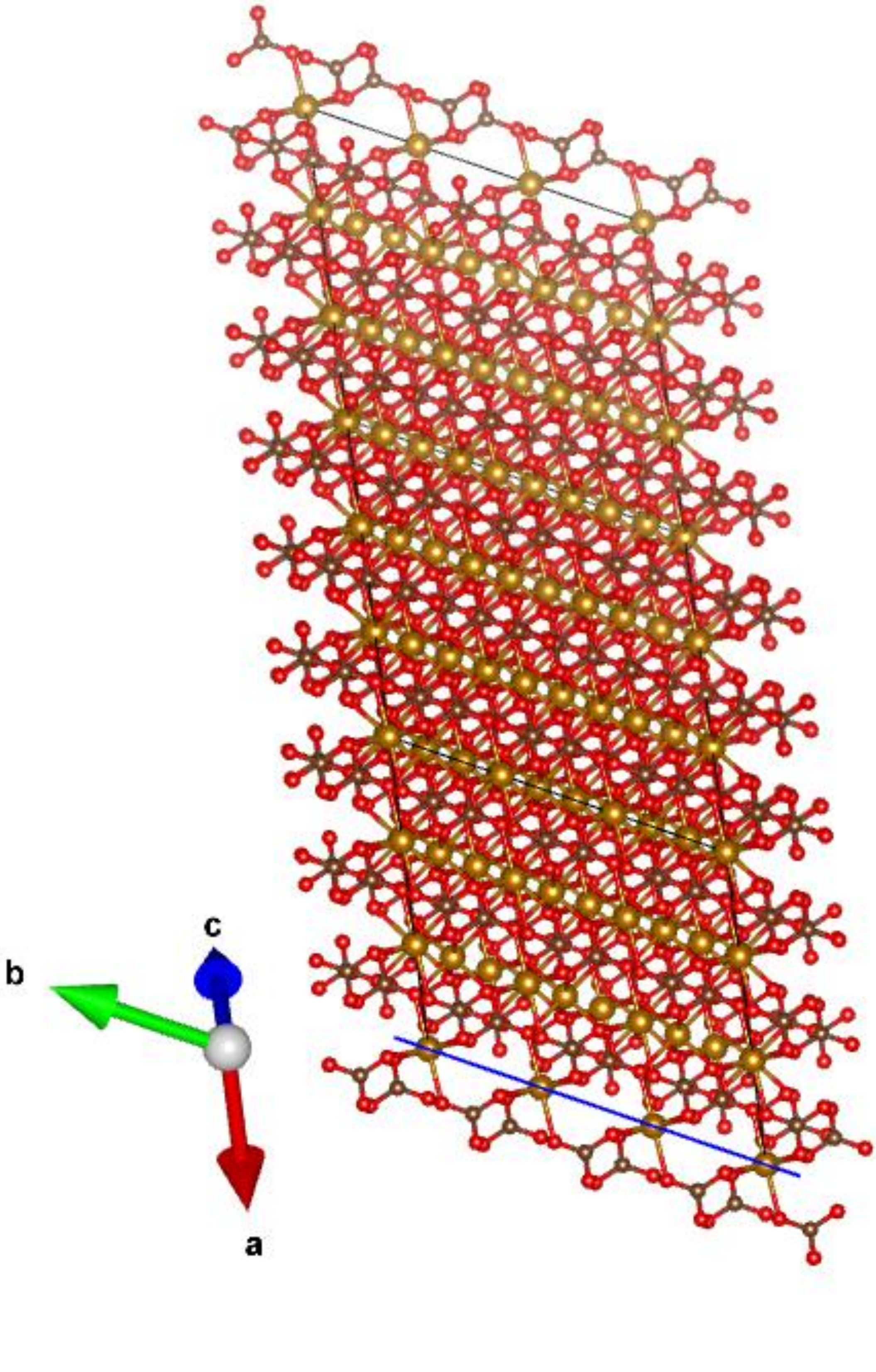

Figure S11. A 2x2x2 until cell of $FeCO3$ showing the [$10\bar{2}$] crystal lattice (blue), which illustrates the Fe atoms of the unit cell with a non-stoichiometric termination.

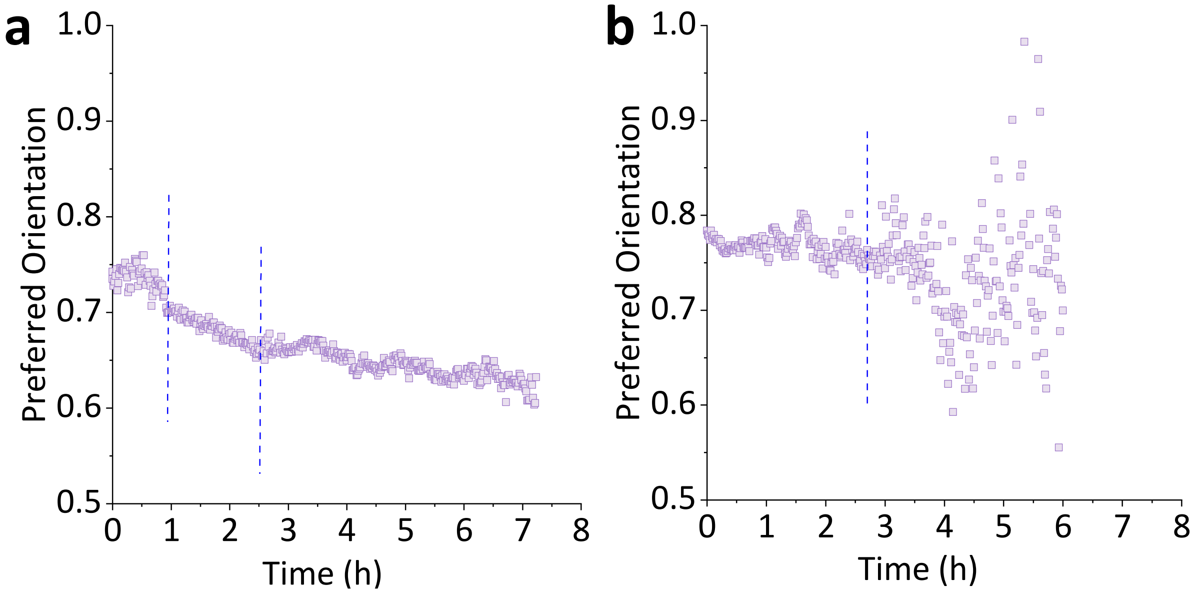

Figure S12. **a)** $FeCO_3$ mild to moderate preferred orientation (along 104) during dissolution at low pH and 25°C. A value of 1.0 corresponds to no preferred orientation; **b)** Mild PO (104) up to ~2.6 hours, after that erratic behaviour from moderate to non-preferred orientation which could be related to dissolution.

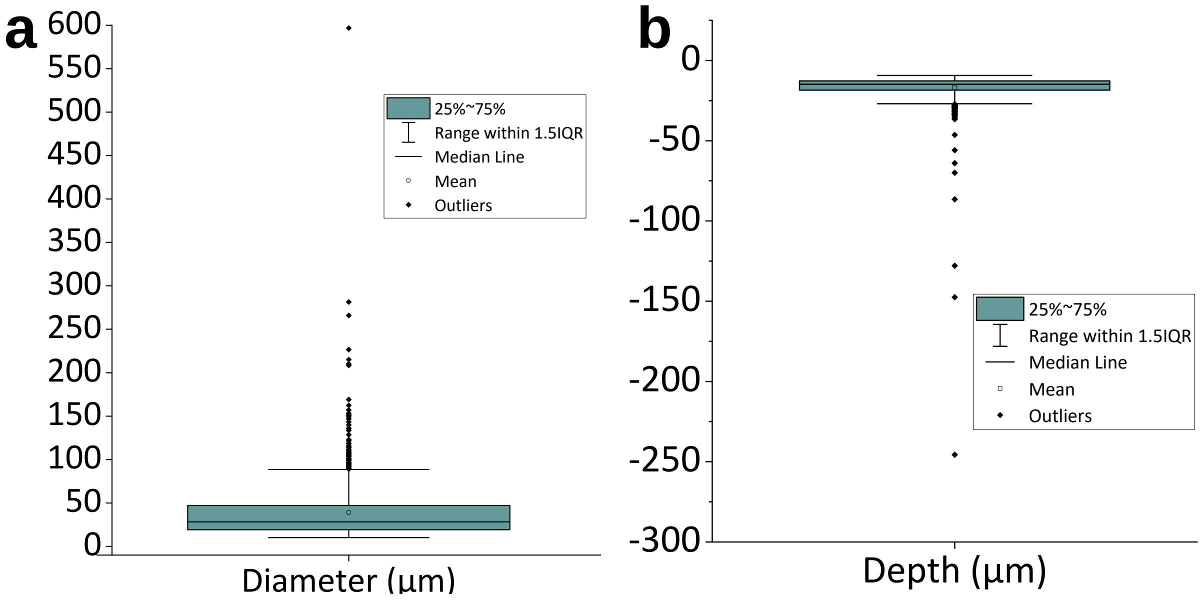

Figure S13 **a)** Diameter (mm), and **b)** depth (mm) of pits quantified after dissolution using 1% HCl flowing at 1 m/s, at pH 3.6 and 80°C using a NPFLEX 3D interferometer (Bruker).

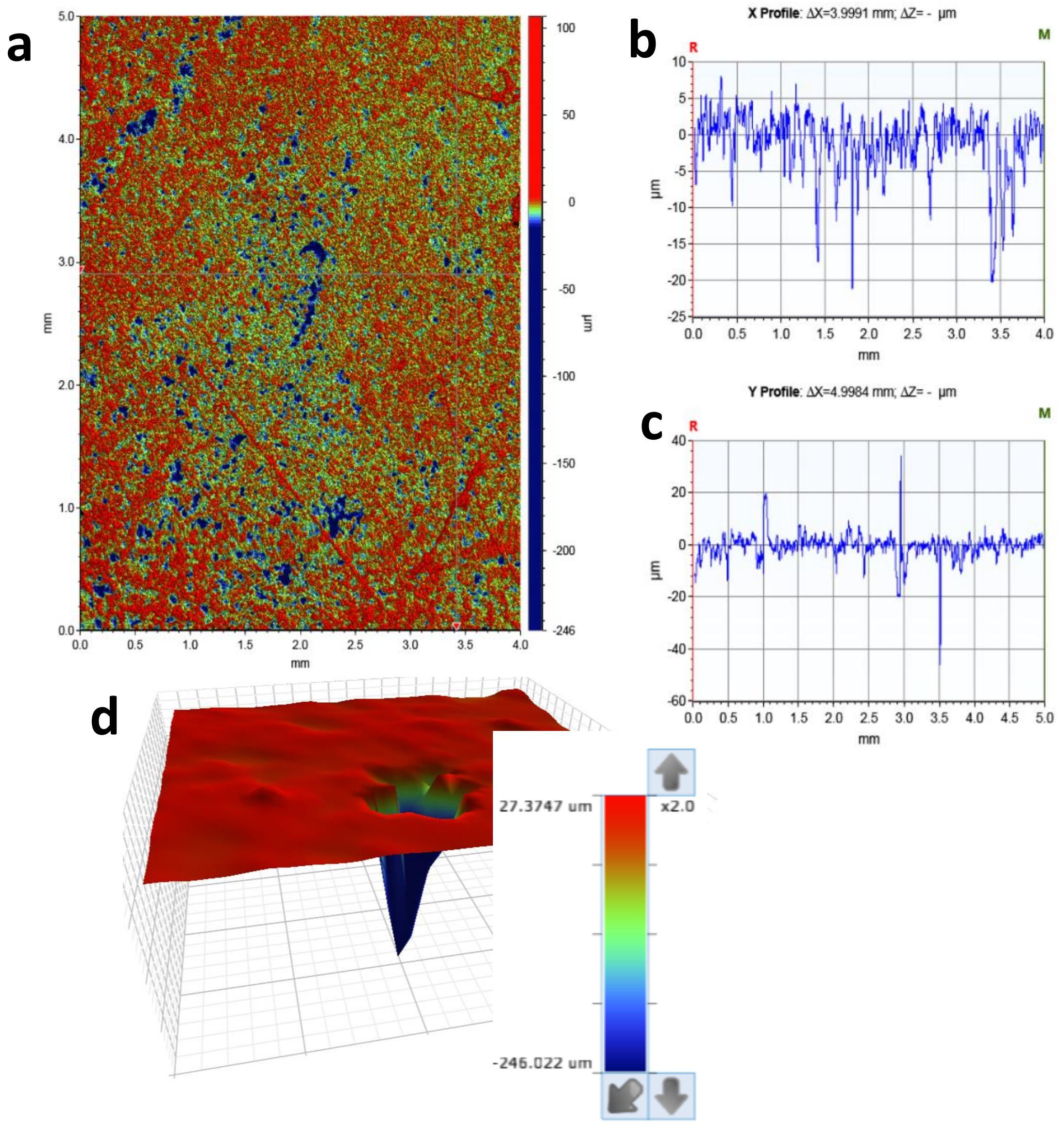

Figure S14. a) Pits mapped (5.0 x 4.0 mm) after dissolution using 1% HCl flowing at 1 m/s, at pH 3.6 and 80°C using a NPFLEX 3D interferometer (Bruker); **b)** showing the diameter profile in mm of the mapped area; **c)** showing the depth profile in mm of the mapped area; **d)** showing a >250 mm pit formed during the reaction demonstrating a significant damage on the steel surface.

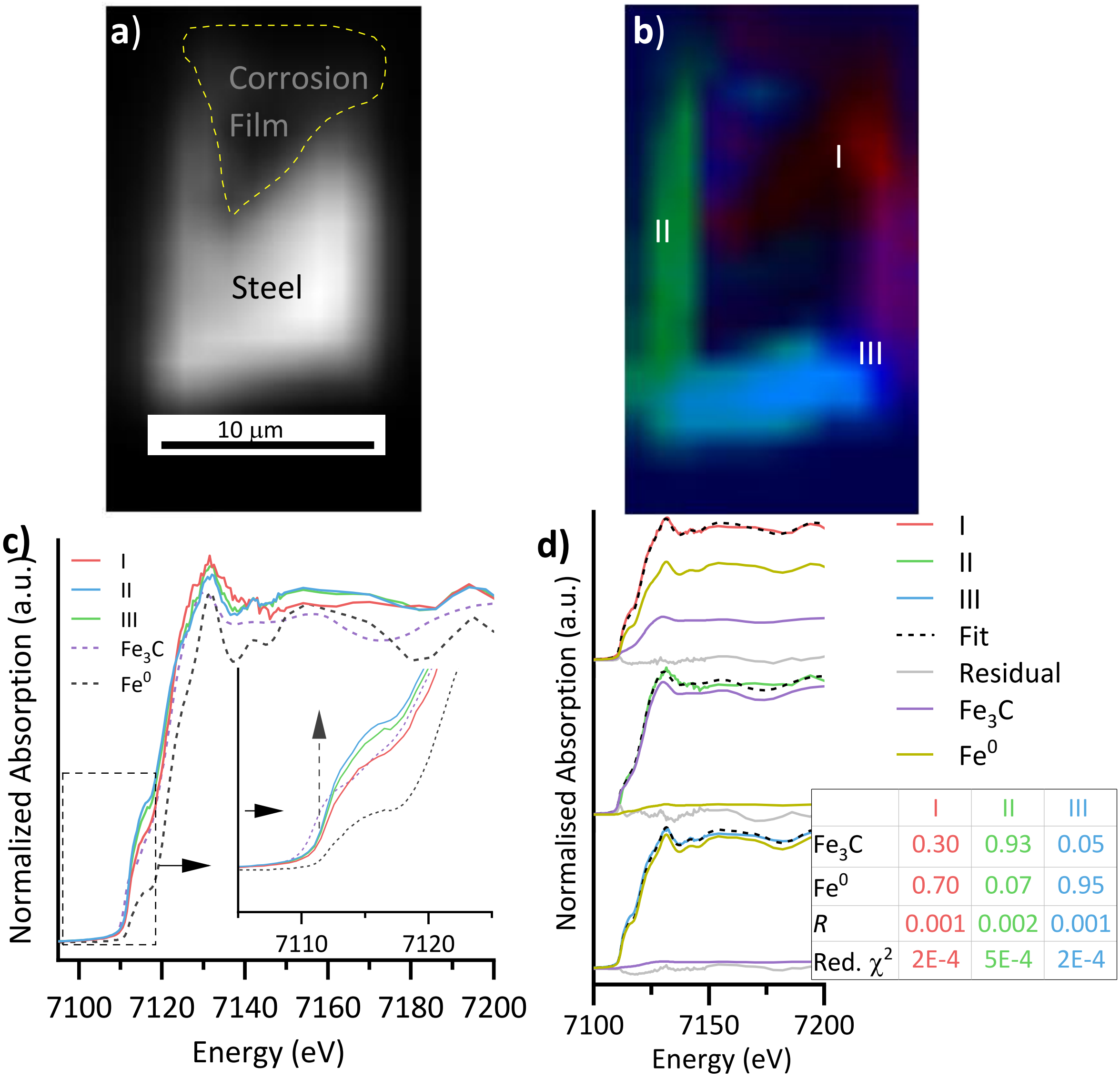

Figure S15. Data from Matamoros-Veloza, A. et al.,2021. **a)** Optical microscope image of the cross section containing the corrosion film (marked area) and steel (area in white) after dissolution experiment at pH 3.6, 80°C using 1% NaCl and 1m/s flow velocity; **b)** XANES cluster map (16 µm x 30 µm) identifying three slightly different Fe local environments; **c)** Fe K-edge XANES spectra I, II and III showing differences in pre-edge intensities and energy shift; **d)** Fe k-edge XANES spectra I, II and III and LCF.

Table S1 Diffraction peaks of the three distinct phases identified on the patterns over the 7.7h dissolution process at room temperature.

**$FeCO_3$**			**$FeCl_2$**			**α–Fe**		
Lattice (hkl)	2q	Q (Å^{-1})	Lattice (hkl)	2q	Q (Å^{-1})	Lattice (hkl)	2q	Q (Å^{-1})
10-2*	2.57	1.8	10-2	3.25	2.3	110	4.56	3.3
104	3.31	2.4	104	3.92	2.8	200	6.45	4.6
202	4.7	3.4	10-5	4.35	3.1	211	7.9	5.6
2-10*	4.95	3.5	2-10	5.19	3.7	220	9.13	6.5
20-4	5.15	3.7	107	5.33	3.8	310	10.21	7.3
10-8	5.32	3.8	202	6.13	4.4	321	12.09	8.6
2-16	6.13	4.4	205	6.42	4.6	330	13.72	9.8
1010	6.43	4.6	2-16	6.77	4.8			
300	6.83	4.9						

Green shading indicates overlap of $FeCO_3$ and $FeCl_2$ peaks while yellow shading indicates. overlap of $FeCO_3$ and Fe peaks.

Table S2 Diffraction peaks of the three distinct phases identified on the patterns over the 6.3 h dissolution process at 80°C.

**$FeCO_3$**			**α–Fe**		
Lattice (hkl)	2q	Q ($Å^{-1}$)	Lattice (hkl)	2q	Q ($Å^{-1}$)
1,0,-2*	2.56	0.18	1,1,0	4.56*	0.32
1,0,4*	3.30	0.23	2,0,0	6.44	0.46
2,-1,0*	3.93	0.28	2,1,1	7.9	0.56
2,-1,3*	4.31	0.31	2,2,0	9.13	0.65
2,0,2	4.68	0.33	3,1,0	10.22	0.73
2,0,-4	5.14	0.37	2,2,2	11.18	0.79
2,-1,6*	5.31	0.38	3,2,1	12.1	0.86
3,-1,-2*	6.11	0.43	4,1,1	13.72	0.97
3,-1,4	6.45	0.46	4,2,0	14.5	1.03
3,0,0	6.81	0.48			
0,0,12	7.20	0.51			
2,0,-10	7.53	0.54			
3,-1,-8	7.70	0.55			
4,-1,-4	8.53	0.61			
3,0,12	9.93	0.71			

Yellow shading indicates overlap of $FeCO_3$ and Fe peaks.

Reference

Matamoros-Veloza, A.; Barker, R.; Vargas, S.; Neville, A., Mechanistic Insights of Dissolution and Mechanical Breakdown of FeCO3 Corrosion Films. ACS Appl. Mat. & Interfaces. **2021**, 13, 5741-5751
